# LAT1-specific PET radiotracers: Development and clinical experiences of a new class of cancer-specific radiopharmaceuticals

**DOI:** 10.7150/thno.99490

**Published:** 2025-01-02

**Authors:** Arifudin Achmad, Hirofumi Hanaoka, Holis Abdul Holik, Keigo Endo, Yoshito Tsushima, Achmad Hussein S. Kartamihardja

**Affiliations:** 1 Department of Nuclear Medicine and Molecular Theranostics, Faculty of Medicine, Universitas Padjadjaran, Bandung 40161, West Java, Indonesia.; 2 Theranostic Radiopharmaceutical Research Collaboration Center, Universitas Padjadjaran, Sumedang 45363, West Java, Indonesia.; 3 Oncology and Stem Cell Study Center, Faculty of Medicine, Universitas Padjadjaran, Bandung 40161, West Java, Indonesia.; 4 Faculty of Medicine, Kansai Medical University, Hirakata 573-1010, Japan.; 5 Department of Radiotheranostics, Gunma University Graduate School of Medicine, Maebashi 3718511, Japan.; 6 Department of Pharmaceutical Analysis and Medicinal Chemistry, Faculty of Pharmacy, Universitas Padjadjaran, Sumedang 45363, West Java, Indonesia.; 7 Kyoto College of Medical Science, Kyoto 6220041, Japan.; 8 Department of Diagnostic Radiology and Nuclear Medicine, Gunma University Graduate School of Medicine, Maebashi 3178511, Gunma, Japan.

**Keywords:** LAT1, PET, radiopharmaceutical, cancer, amino acid metabolism

## Abstract

The quest for a cancer-specific positron emission tomography (PET) tracer has been ongoing for decades. Current evidence shows that targeting amino acid metabolism dysregulation is a valid alternative cancer detection method and can complement the conventional approach, which relies on targeting increased glucose metabolism. The rate of amino acid metabolism in all major organs is mostly equally low and does not change in any physiological dynamics. The amino acid metabolism rate only spikes in malignant tissues. PET imaging targeting LAT1 (L-type amino acid transporter 1) demonstrated accurate cancer imaging of various cancer types with nearly negligible background uptake. LAT1 is a *pan-cancer* biomarker of amino acid metabolism dysregulation. The upregulated LAT1 expression in cancer cells depicts their dynamic behavior and aggressiveness. This review discussed PET radiotracers developed as a LAT1-specific agent and how this new class of cancer-specific radiopharmaceuticals could deliver PET images with clinical properties we yearn for, such as high specificity toward various malignancies, robust non-cancer exclusion (mainly inflammatory reactions), accurate malignant lesion delineation, representative therapeutic monitoring, and long-term prognostication.

## Introduction

Positron emission tomography (PET) has existed for about a half-century, yet a true oncologic PET radiotracer with *pan-cancer* efficacy remains elusive. The 2-deoxy-2-[^18^F]fluoro-D-glucose ([^18^F]FDG) has been the dominant PET radiotracer for oncology since PET invention due to its high sensitivity for malignancies with enhanced glycolysis 1. By 2015, [^18^F]FDG PET is indicated in the evidence-based guidelines for six cancer types (lung, colorectal, lymphoma, head and neck, melanoma, and esophageal cancer) 2 and only recently for pediatric oncology (mainly lymphomas and sarcomas) and ovarian cancer 3, 4. However, the cancer-specificity of [^18^F]FDG is often compromised in slow-growing tumors, lesions adjacent to inflammations, or lesions located within organs with high-rate glycolysis, such as the brain and liver 1. Thus, in nuclear neurooncology, [^18^F]FDG is less useful than radiolabeled amino acids, which are more suitable for wider clinical scenarios 5, 6.

Since the early days of [^18^F]FDG, hundreds of PET radiotracers that targeted oncologic molecular biomarkers such as metabolic dysregulation (amino acid, fatty acids, or lactate), cellular proliferation (synthesis of proteins, DNA, and cell membranes) or expression of various receptors, enzymes, and tumor-associated or tumor-specific antigens have been investigated 7. However, only a handful have obtained regulatory approval for oncology and are routinely used worldwide: [^68^Ga]Ga-DOTATATE, [^68^Ga]Ga-PSMA, [^18^F]NaF, [^18^F]FDOPA, and [^18^F]FLT 8. Recently, we witnessed a breakthrough as fibroblast activation protein (FAP) inhibitor (FAPI)-based PET radiotracers being used to target cancer-associated fibroblasts in the stroma of desmoplastic solid tumors. In most cases, FAPI delivers a high-contrast image 9. However, FAP is also expressed in chronic inflammations, benign tumors, and fibrosis 10. FAPI is also less superior than [^18^F]FDG in lymphoma and multiple myeloma 11, 12. Despite their unique properties, none of these PET radiotracers have had the versatility to compete with [^18^F]FDG as a general oncologic PET radiotracer 8.

L-type amino acid transporter 1 (LAT1) has been regarded as a *pan-cancer*, cancer-specific molecular target 13. The drug discovery for LAT1 has been going on for years and has narrowed to a few compounds. We reviewed the development, application, and future potential of PET radiopharmaceuticals that have been proven or claimed as LAT1-specific PET radiotracers.

## Choosing targets and tracer design for *pan-cancer*, cancer-specific PET radiotracer

Molecular target selection is paramount in designing *pan-cancer* PET radiotracers. The target should be overexpressed in various cancers and have negligible physiologic expression. The target should also be readily accessible for tracer uptake, thus often a cell surface protein. It has to be robust from enzymatic degradation, has a low turnover rate, and is not shed from the cells nor trapped within the cells 14. On the other side, the PET radiotracer should have high specificity and affinity for the target, along with low/negligible nonspecific binding toward normal tissues and non-cancer pathologies. It should also be sufficiently hydrophilic for rapid and large-volume distribution throughout the body and fast plasma clearance 14. Small molecules fit this purpose, and their structure often allows for ^18^F radiolabeling suitable for routine PET use 15.

The upregulated amino acid transporters in cancer have long been attractive targets 16. Four transporters stand out as potential *pan-cancer* targets: LAT1 (L-type amino acid transporter 1), ASCT2 (Alanine, Serine, Cysteine Transporter 2), SNAT1, and SNAT2 (Sodium-coupled Neutral Amino Acid Transporter 1 and 2) 13. Their overexpression in multiple cancer types is summarized in **Table S1** (ranked according to the global cancer burden data from GLOBOCAN 2022) 17). LAT1 (encoded by the SLC7A5 gene) is a transmembrane protein, a member of the system-L amino acid transporter family (LAT1-4). In some cancers, LAT1 expression increases along with the increase of ASCT2 expression. However, ASCT2 is physiologically expressed in normal tissues of the lungs, muscles, large intestine 18, and inflammatory T cells 19, making ASCT2 less ideal as a cancer molecular target. In contrast, the LAT1 protein expression level on the plasma membrane of normal tissue is significantly lower than in cancer cells 20, 21. In some cancers, e.g., ovarian epithelial carcinoma, the LAT1 mRNA expression level may be up to 10-29 times higher than the normal cells, resulting in significantly higher LAT1 protein expression 22. LAT1 protein overexpression is consistent across diverse cancers, including breast, lung, stomach, colorectal, liver, esophagus, pancreas, kidney, brain, ovarian cancer 23, and non-Hodgkin's lymphoma 24. LAT1 expression is also intertwined with various cancer hallmarks, and, more importantly, no evidence to date that LAT1 expression is involved in tumor-promoting inflammation in the cancer microenvironment 25. Therapeutic studies in various cancer cells and xenografts showed that LAT1-specific inhibition halts cancer proliferation 26.

## Challenges in designing LAT1-targeting PET radiotracer

The main requirements for LAT1-targeting radiotracers are high affinity and selectivity to LAT1. They should not inhibit other amino acid transporters expressed in non-cancer cells or other pathologies. In fact, unlike glucose or fatty acid transport systems, the amino acid transport system is way more complex. Each amino acid transport system has heterogeneous substrate and transport mechanisms. On top of that, most amino acids are transported by more than one transporter 13, 20. Thus, it is not surprising that almost all amino acid-based PET radiotracers lack selectivity toward a particular transport system, let alone a specific amino acid transporter (**Table 1**). The diagnostic performance in various clinical scenarios is detailed elsewhere 27.

The amino acid transport mechanism is often *bi-directional* (*influx* of one amino acid is mandatory coupled with *efflux* of another amino acid) 28. Thus, using radiolabeled amino acids plainly as PET radiotracers is challenging just by relying on their substrate characteristics. Bi-directional transport leads to a high washout rate; consequently, tumor uptake and retention may not be adequate, compromising its sensitivity. On the other hand, compounds that have a long history of LAT1-selective substrate or inhibitors (e.g., melphalan, gabapentin, T3 hormone) do not always have a strong LAT1 affinity 20.

**Figure 1:**
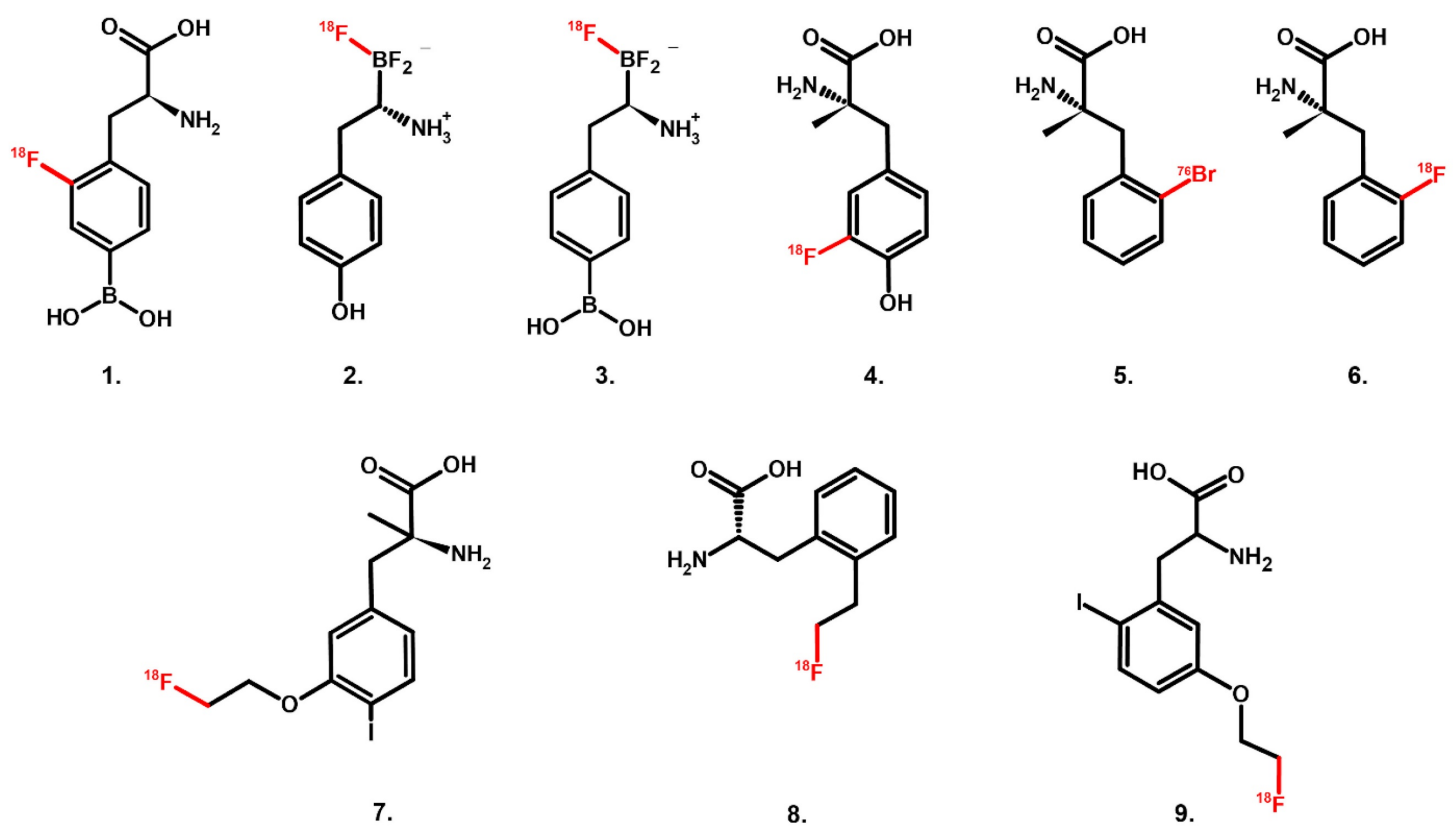
Chemical structure of PET radiotracers with high selectivity towards LAT1; **1.** [^18^F]FBPA, **2.** [^18^F]FBY, **3.** [^18^F]BBPA, **4.** [^18^F]FAMT, **5.** [^76^Br]BAMP, **6.** [^18^F]FAMP, **7.** [^18^F]FIMP, and **8.** [^18^F]FELP, and **9.** [^18^F]NKO-035.

The requirement for LAT1 recognition and the spectrum from substrate-type toward inhibitor-type of LAT1-selective compounds have been roughly mapped. A LAT1 substrate should possess at least an intact amino group, a carbonyl oxygen and alkoxy oxygen of the carboxyl group, and a hydrophobic side chain. Adding an α-methyl group into aromatic amino acids renders a compound a LAT1-selective substrate at the price of decreasing its LAT1 affinity. On the other end of the spectrum, high-affinity LAT1 inhibitors should have an α-amino acid structure with free carboxyl and amino groups and a hydrophobic bulky side chain, where the carbonyl oxygen of the carboxyl group should not be involved in intramolecular hydrogen bonding. Adding O-linked hydrophobic bulky moiety at the *para*-position of the aromatic ring is suggested as crucial to retaining LAT1-selectivity while having high affinity. However, hydrophobic moieties can sometimes remove the LAT1-selectivity entirely 20. The LAT1-specific PET radiotracers discussed here mostly belong to the substrate-type compound (**Figure 1**). Phenylalanine has long been known as a LAT1 high-affinity substrate (*K*_m_ = 14.2 *µ*M) 52. In order to develop an ideal LAT1-specific PET radiotracer based on LAT1 substrates, plasma amino acid concentrations should also be considered 53 (See **Graphical Abstract**). Unsurprisingly, phenylalanine is the common backbone structure of these LAT1-specific radiotracers 54.

## LAT1-specific PET radiotracers

### 1. [^18^F]FBPA, [^18^F]FBY, and [^18^F]BBPA

The 4-borono-2-[^18^F]fluoro-D,L-phenylalanine ([^18^F]FBPA) was first synthesized in 1990, long before LAT1 was discovered 55. [^18^F]FBPA is a surrogate marker for tumor accumulation of 4-borono-D,L-phenylalanine (BPA). The boron atom (^10^B) of BPA is the target compound of boron neutron capture therapy (BNCT), an irradiation technique for unresectable local malignancies. BPA is administered intravenously and ultimately accumulates within cancer cells. When bombarded by neutrons, ^10^B atoms turn into radioactive ions radiating alpha particles, killing those cancer cells 56. By radiolabeling BPA with ^18^F, [^18^F]FBPA PET study prior to BNCT can approximate the tumor-to-normal tissue (T/N) accumulation ratio of BPA (^10^B). The T/N ratio was defined as the ratio of SUV_max_ obtained from tumor ROI to the SUV_max_ of the ROI of normal tissue surrounding the tumor lesion at the corresponding level on the PET/CT image slice. Several clinical trials showed that tumor lesions with a T/N ratio ≥ 2.5 are eligible for BNCT 57.

[^18^F]FBPA is a product of ^18^F radiolabeling either via [^18^F]F_2_ electrophilic substitution or [^18^F]F^-^ nucleophilic substitution (typical yield in both approaches was similarly low, ±20-25%) 57. A recent nucleophilic method produced non-carrier-added [^18^F]FBPA with acceptable molar activity (56 ± 15 GBq/*μ*mol) and suggested the potential to be automated for routine use 58. It is worth noting that two isomers exist, L-[^18^F]FBPA and D-[^18^F]FBPA, depending on the radiolabeling starting material. Even though L-[^18^F]FBPA has been chosen since early clinical use, the consideration was not LAT1 selectivity but the preference of melanoma for taking up L-enantiomers of amino acids 59. On the other hand, D-amino acids are known for their lack of contribution to human amino acid metabolism. Indeed, D-[^18^F]FBPA has scant accumulation in the brain, liver, spleen, pancreas, and bones 60. Unfortunately, D-[^18^F]FBPA is neither selective to LAT1 nor LAT2, nullifying its potential as a cancer-targeting PET radiotracer 61. The term “[^18^F]FBPA” in this review is meant for L-[^18^F]FBPA.

**Figure 2:**
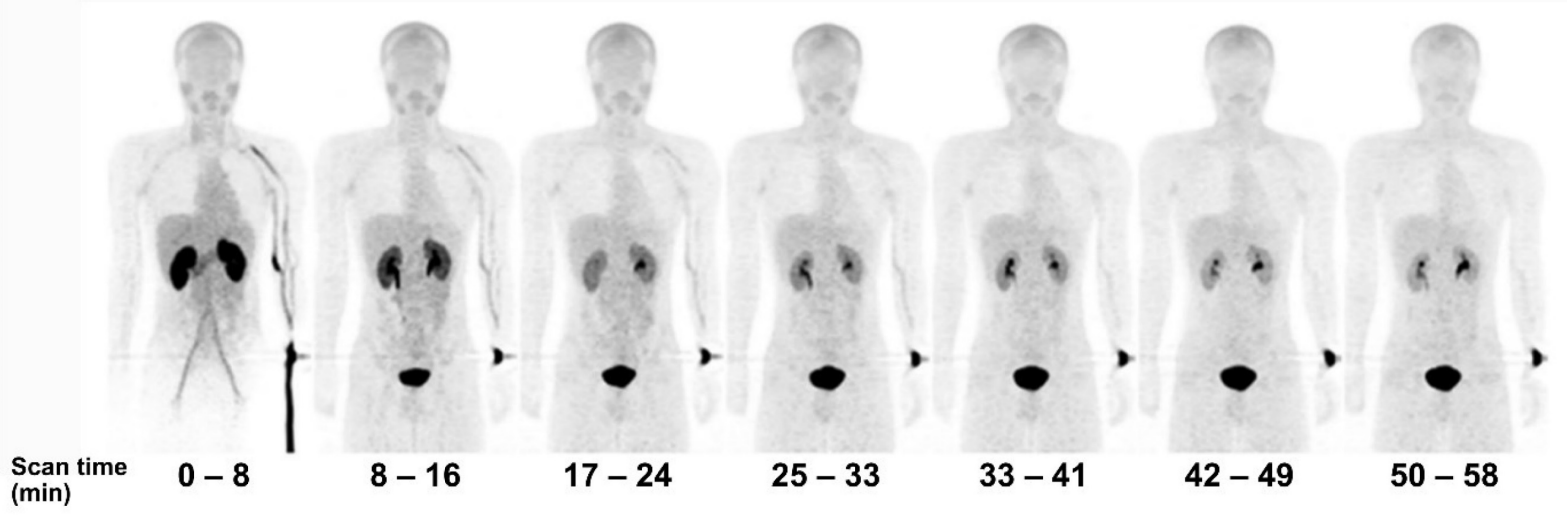
Serial PET mean intensity projection (MIP) images of [^18^F]FBPA uptake in a healthy male. Kidney and pancreas uptake [^18^F]FBPA rapidly cleared after injection. Pelvic kidney and bladder accumulation represent [^18^F]FBPA excretion route. A high-contrast image can be obtained less than 1 hour after injection 65. Adapted and reproduced with permission from Springer Nature. Copyright © 2016, The Japanese Society of Nuclear Medicine.

**Figure 3:**
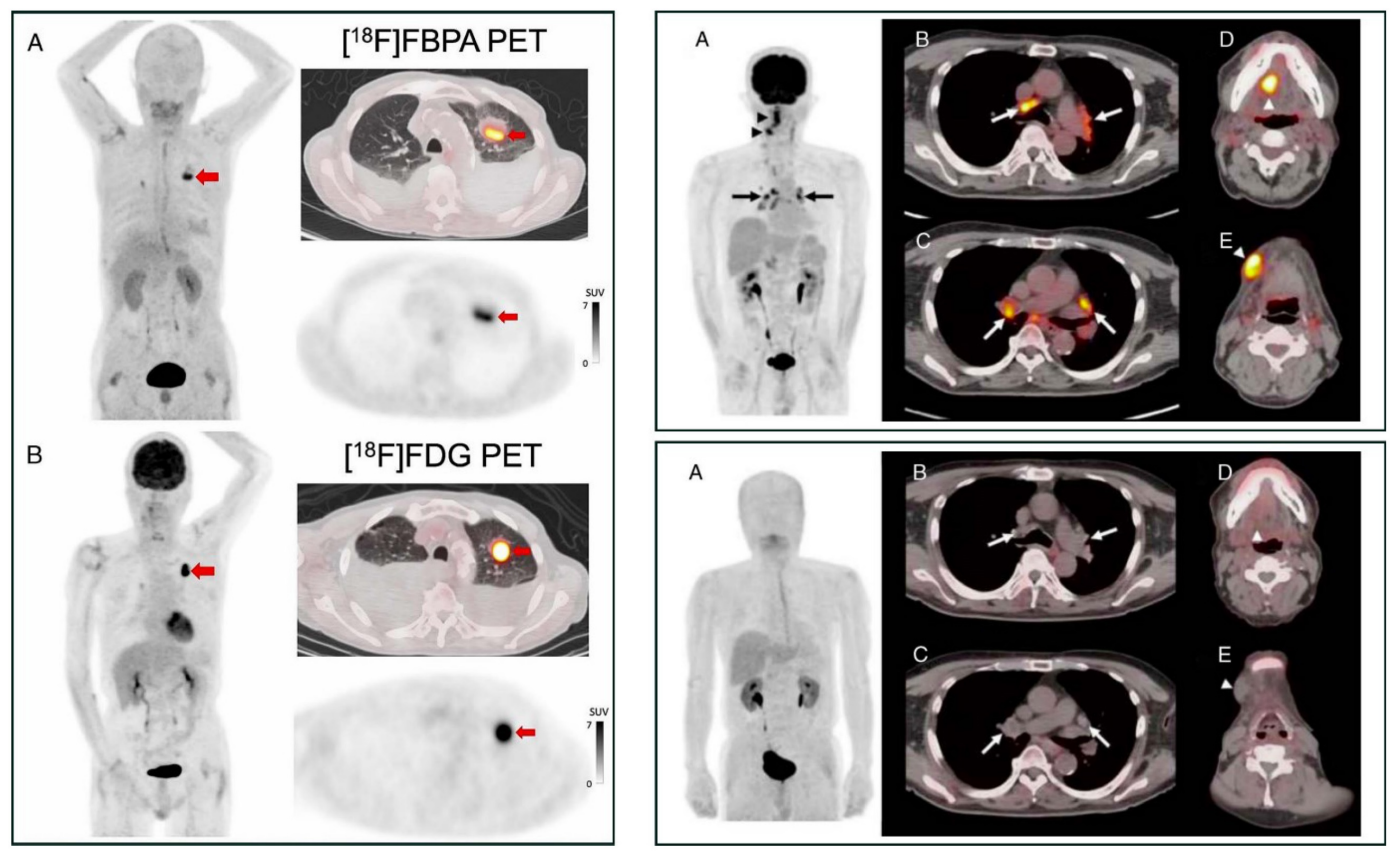
PET MIP and axial PET/CT image comparison between [^18^F]FBPA and [^18^F]FDG. **Left box:** A primary lung squamous cell carcinoma on a 71-year-old man (*red arrows*, [^18^F]FDG SUV_max_ 14.8 and [^18^F]FBPA SUV_max_ 7.2). Reproduced as is from 66. Copyright © 2023, Copyright © 2023 The Author(s). Published by Wolters Kluwer Health, Inc. Originally published under Creative Commons Attribution-Non Commercial-No Derivatives License 4.0 (CCBY-NC-ND license). **Right boxes:** A tongue cancer and mediastinal sarcoidosis case in a 68-year-old man. The [^18^F]FDG PET images ***(upper box)*** and [^18^F]FBPA PET images ***(lower box)*** were taken ten and eleven days following high-dose brachytherapy for his tongue cancer, respectively. [^18^F]FDG PET images visualized active inflammation in his mediastinal sarcoidosis **(*upper box*, A-C, arrows)** and post-radiation inflammation **(*upper box*, D & E, arrowheads)**, while no [^18^F]FBPA uptake in the corresponding area of the mediastinum **(*lower box*, A-C, arrows)**, tongue, and submandibular region **(*lower box*, D & E, arrowheads)**. Reproduced as is from 67. Copyright © 2020, Copyright © 2020 The Author(s). Published by Wolters Kluwer Health, Inc. Originally published under Creative Commons Attribution-Non Commercial-No Derivatives License 4.0 (CCBY-NC-ND license).

[^18^F]FBPA is claimed as LAT1-specific (*K*_m_ LAT1 = 197 *µ*M, *K*_m_ ratio LAT2/LAT1=14), while BPA has much lower LAT1-specificity (*K*_m_ ratio LAT2/LAT1 = 4.3) 62. In melanoma, [^18^F]FBPA is involved in melanogenesis, and thus melanoma lesions have higher [^18^F]FBPA uptake 57. Adding the α-methyl group might improve the LAT1 selectivity (this tracer is still in early investigation) 63. As a LAT1 substrate (subject to bi-directional transport), consequently, [^18^F]FBPA tumor uptake is generally lower than PET radiotracers whose trapping mechanism within cancer cells, like [^18^F]FDG. However, [^18^F]FBPA tumor uptake is about similar to that of [^11^C]MET 64. A high-contrast image in [^18^F]FBPA PET can be obtained earlier than the typical 60 min post-injection scan (**Figure 2**) 65. [^18^F]FBPA PET visualized thoracal and mediastinal malignant lesions with high contrast (**Figure 3A**) 66. In benign lesions, [^18^F]FBPA uptake was consistently low (SUV_max_ < 2), while [^18^F]FDG uptake on the same lesions was higher (SUV_max_ > 5). More importantly, unlike [^11^C]MET and [^18^F]FDG, [^18^F]FBPA does not accumulate in inflammatory tissues, including post-radiation necrotic lesions (**Figure 3B**) 67.

Head-to-head comparisons between [^18^F]FBPA and [^18^F]FDG PET involving enough patients are scarce. The SUV_max_ of [^18^F]FBPA and [^18^F]FDG are strongly correlated (n = 20, *r* = 0.72) for detecting head and neck cancer lesions 68. However, their SUV_max_ correlation is weak (*r* = 0.48) as reported by a study (n = 82) involving head and neck squamous cell carcinoma and adenocarcinoma, adult and juvenile type sarcoma, malignant melanoma, and glioma) 69. Interestingly, their intratumoral spatial distribution—measured by spatial parameters such as metabolic tumor volume (MTV)—is significantly dissimilar 70. Even so, the absence or limited uptake of [^18^F]FBPA in benign lesions might be favorable for accurate cancer detection. In an exploratory study (n = 22 primary tumors, 55 metastatic lesions, and 11 benign lesions), Isohashi *et al.* found that the maximum standardized uptake volume (SUV_max_) of [^18^F]FBPA is significantly higher for malignant lesions (5.1 ± 3.0 vs. 2.9 ± 0.6, *p* < 0.001), resulting in a potentially useful SUV_max_ cutoff at 3.24. In the same cohort, the SUV_max_ of [^18^F]FDG could not adequately discriminate malignant and benign lesions (SUV_max_ of 11.16 yields 90% sensitivity but only 39% specificity) 71. However, in head and neck cancer cases, the PET parameters (SUV_max_, SUV_peak_) of [^18^F]FBPA were weakly correlated with LAT1 expression in tumor tissue specimens. Thus, sometimes [^18^F]FBPA uptake could not be reliably predicted BNCT treatment response 72.

**Figure 4:**
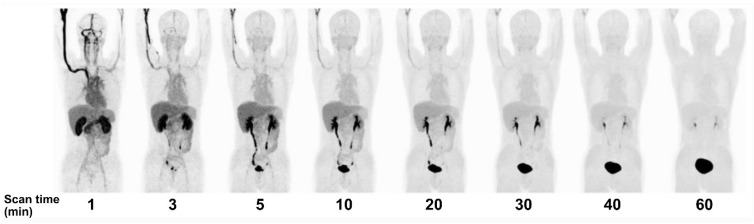
PET MIP images over time after [^18^F]FBY administration in a healthy volunteer. Adapted with permission from 74. Copyright © 2021, The Author(s), under exclusive license to Springer-Verlag GmbH, DE, part of Springer Nature.

Since June 2020, BNCT has been covered by national insurance in Japan for head and neck cancer. Thus, we may expect a wider use of [^18^F]FBPA PET. Meanwhile, Li *et al.* in China developed [^18^F]trifluoroboratetyrosine ([^18^F]FBY) as an alternative for BPA and [^18^F]FBPA (**Figure 1**) 73. The reason was twofold: 1) *in vivo* deboronation of [^18^F]FBPA and 2) the distinct molecular structure between BPA and [^18^F]FBPA due to additional [^18^F] on the [^18^F]FBPA side chain. Both reasons contributed to the off-target irradiation during BNCT. The [^18^F]FBY unique radiolabeling approach replaces the amino acid carboxyl group with a negatively charged trifluoroborate group, allowing for quick radiolabeling (15 min) with favorable yield (~50%), radiochemical purity (~98%), and stability (4 h). [^18^F]FBY has molecular electrostatic potential, structure, and chemical characteristics similar to BPA and is metabolically more stable than BPA. Despite no information regarding LAT1 selectivity (in comparison to LAT2), their *in vitro* and animal studies showed that boron concentration in tumor, muscle, and brain (therapeutic dose of FBY) is highly correlated with the mean SUV of [^18^F]FBY in those regions. In humans, [^18^F]FBY PET images presented a rapid kidney clearance, resulting in an extremely low background uptake at 60 min post-injection (**Figure 4**) 74. Preliminary PET studies in primary brain tumors and brain metastases showed very promising potential of [^18^F]FBY (T/N ratio at least 17.1 and 20.2, respectively 75 and better brain tumor lesions delineation than contrast-enhanced MRI 76).

Li *et al*. continued their elaboration, and recently, they successfully synthesized [^18^F]trifluoroborate-derived boronophenylalanine ([^18^F]BBPA, **Figure 1**). The synthesis method was able to yield a stable and more than enough final [^18^F]BBPA activity (± 1 Ci) for routine clinical PET imaging, and [^18^F]BBPA was also stable and potentially a better BNCT compound since it has more boron atom and more tumor accumulation than BPA. [^18^F]BBPA showed excellent pharmacokinetics and provided a clean background for high-contrast PET images. A limited clinical PET study showed that [^18^F]BBPA may reach a T/N ratio in the brain up to 18, higher than most amino acid PET radiotracers 77. However, more clinical data is warranted since [^18^F]BBPA was also reported to accumulate in non-malignant tissue 78.

### 2. [^18^F]FAMT and [^18^F]FAMP

Among LAT1-specific amino acid-based PET radiotracers, 3-[^18^F]fluoro-α-methyl-L-tyrosine ([^18^F]FAMT, **Figure 1**) is probably the most extensively studied clinically for cancer within the last two decades 20. The history of α-methyl-L-tyrosine compounds predates back to the 1980s when 3-[^123^I]iodo-α-methyl-L-tyrosine ([^123^I]IMT) SPECT was used as an alternative for [^11^C]MET PET imaging of brain tumors 79. It was revealed a decade later that [^123^I]IMT is LAT1-selective 80, although it was also revealed that [^123^I]IMT is a substrate for B^0^ amino acid transporter system 81. Compared to [^11^C]MET PET for routine clinical use (^11^C half-life is very short, and in the 1990s, PET camera was a rarity), [^123^I]IMT SPECT was superior. However, the [^123^I]IMT SPECT spatial image resolution is much lower than [^11^C]MET PET, precluding the detection of small lesions 79.

In 1997, Tomiyoshi *et al.* at Gunma University developed a quick method (43 min) to synthesize [^18^F]FAMT via [^18^F]F_2_ gas electrophilic substitution of α-methyl-L-tyrosine (radiochemical yield ± 20%, final radioactivity obtained was ± 1.5 GBq (40 mCi), limiting its use only to 2-4 patients) 82. The imaging comparison in breast cancer xenografts with [^11^C]MET, [^99m^Tc]Tc-tetrofosmin, [^99m^Tc]Tc-sestamibi, and [^201^Tl]Tl-chloride showed that [^18^F]FAMT had the highest tumor uptake, tumor-to muscle, and tumor to lung ratio 83. Despite the low yield and low specific activity in each radiosynthesis, [^18^F]FAMT has been routinely used in PET clinical studies until now. An alternative synthesis route for [^18^F]FAMT was proposed years later via an automated, three-step, two-pot procedure of nucleophilic exchange reaction producing 32 ± 8% radiochemical yield (140 min) with a suitable amount of radioactivity for clinical use (> 20 GBq/mmol) 84. The current automated synthesis could produce [^18^F]FAMT up to 39 GBq/mmol specific activity 85. One of the late-stage fluorination methods, which has been popular recently, has the potential to synthesize [^18^F]FAMT with final radioactivity per batch of at least 10 GBq (270 mCi)) 86.

**Figure 5:**
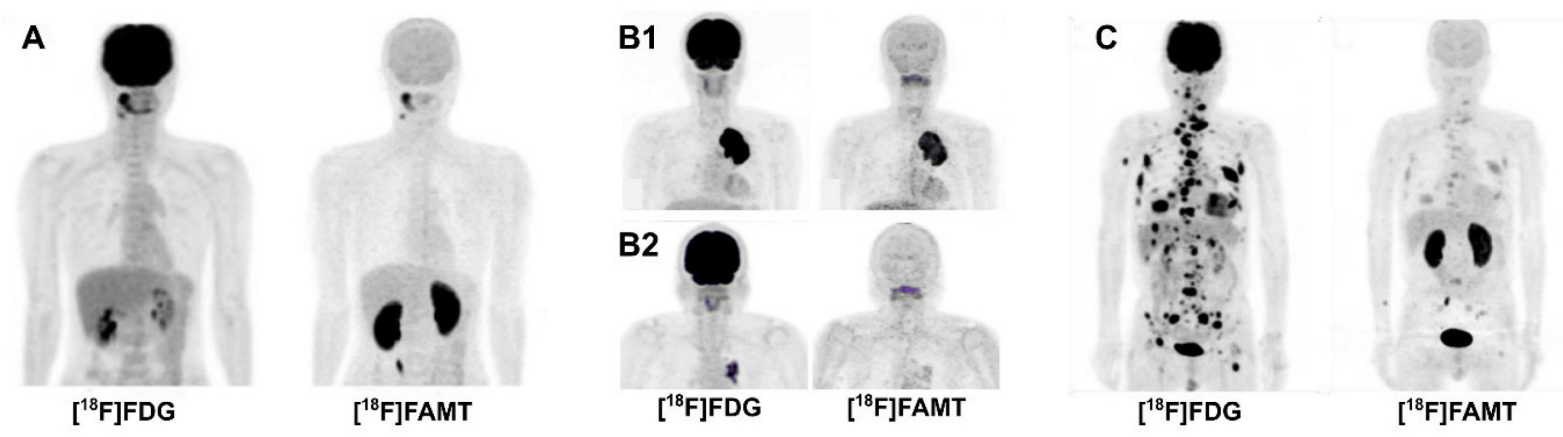
Comparison of PET MIP images of [^18^F]FDG and [^18^F]FAMT in malignancies, 60 min post-administration (*author's data*). **(A)** A 40-year-old man with oral squamous cell carcinoma. [^18^F]FAMT uptake in the brain and other major organs is minimal, while kidney uptake is prominent. **(B1, B2)** [^18^F]FAMT provided a prognostic tool for overall survival in non-small cell lung cancer patients. A 60-year-old man (stage IIIB, T4N0M0) with high [^18^F]FAMT tumor uptake had survived for only 122 days **(B1)**, while a 72-year-old man (stage IIIA, T2N2M0) with faint [^18^F]FAMT tumor uptake indicating less aggressive tumor, survived until 1,875 days later **(B2)**. **(C)** A 73-year-old man with metastatic large-cell neuroendocrine lung carcinoma with faint [^18^F]FAMT uptake in all lesions.

[^18^F]FAMT is a highly specific LAT1-targeting radiopharmaceutical, thanks to its possession of α-methyl group (*K*_m_ LAT1 72.7 *μ*M and no transport via LAT2) 29. [^18^F]FAMT is not metabolized nor incorporated into protein; thus, physiological uptake is negligible. Clinical [^18^F]FAMT PET studies paired with histopathological data in various cases confirmed the presumption that LAT1 expression in normal tissues is very low. Except in the kidney, [^18^F]FAMT physiological uptake in the brain, heart, lung, liver, and bone marrow is significantly less than [^18^F]FDG (**Figure 5**). Most importantly, [^18^F]FAMT uptake in muscle is as low as [^18^F]FDG 50. Thus, even though the absolute lesion uptake of [^18^F]FAMT is lower than that of [^18^F]FDG, high-contrast images can be obtained due to low background activity. [^18^F]FAMT enantiomeric tracer, D-[^18^F]FAMT, has been predicted to have lower renal retention since D-amino acid is rarely involved in human metabolism. The presumption was proven true; however, D-[^18^F]FAMT has even lower absolute tumor uptake than L-[^18^F]FAMT, preventing this enantiomeric tracer from being further explored 87.

[^18^F]FAMT uptake in inflammations is also lower than that of [^18^F]FDG, as confirmed in preclinical 88, 89 and clinical studies 90, 91. A dynamic study in animal tumor models showed that both total distribution volumes of [^18^F]FBPA and [^18^F]FAMT within the tumor represent the tumor expression levels of LAT1 and are not influenced by the tumor blood flow 85. This finding showed [^18^F]FBPA and [^18^F]FAMT potential for evaluating the LAT1 expressions in hypoxic cancer lesions or those with low vascular density. Unfortunately, direct clinical comparison with other amino acid PET radiotracers is lacking. The typical highest major organ uptake of other amino acid PET radiotracers compared to that of [^18^F]FAMT was summarized in **Table 1**.

Head-to-head diagnostic accuracy tests comparing [^18^F]FAMT and [^18^F]FDG for malignancy detection suggested that they have equal sensitivity, yet [^18^F]FAMT has higher tumor specificity 51. [^18^F]FAMT PET/CT has been clinically useful as a diagnostic and prognostic tool in various cancer types, e.g., glioma 92, lung 93, esophageal 94, and oral cancers 95. Clinical [^18^F]FAMT PET studies on various cancers (in comparison to [^18^F]FDG) over the last two decades are summarized in **Table S2**.

The high [^18^F]FAMT radioactivity in the kidney is not only due to the excretory route (**Figure 5**) 50. The 3‑fluorine and 4-hydroxyl group of [^18^F]FAMT render it recognized as a substrate by OAT1, an organic anion transporter on renal tubular epithelial cells, facilitating prolonged kidney retention 96. Administering OAT1 inhibitors (e.g., probenecid) before [^18^F]FAMT injection has been proven efficient in animal models to delay [^18^F]FAMT blood clearance and thus increase tumor uptake 97.

The [^18^F]FAMT absolute tumor uptake is relatively low compared to that of [^18^F]FDG due to the LAT1 bi-directional transport, resulting in fast clearance from the tumor. This quick tumor washout may lead to false negative findings. However, it is important to note that in all [^18^F]FAMT PET clinical studies, a scan is obtained about 60 min post-injection. Therefore, a dynamic [^18^F]FAMT PET study is essential to determine the optimal scan time. A dynamic PET study in mice showed that [^18^F]FAMT can rule out granuloma from gliomas in which static images failed to do so 89. However, glioma uptake patterns might vary for different grades since low-grade glioma metabolizes fewer amino acids and expresses less LAT1 98. Dynamic [^18^F]FET PET studies in glioma provided insights for scan protocol adjustment, thus included in the current guidelines 5. If a dynamic PET study is not feasible, an early static scan at 5-15 min post-injection is more accurate for differentiating glioma grades than the 20-40 static scan 99. Unfortunately, such clinical dynamic PET studies are currently lacking for [^18^F]FAMT.

The limitations in [^18^F]FAMT synthesis and the short half-life of ^18^F inspiring the use of a longer half-life positron emitters, ^76^Br (mean energy 650 keV, *t*_1/2_ = 16.2 h) and ^77^Br (mean energy 336 keV, *t*_1/2_ = 57 h) for α-methyl-L-tyrosine radiolabeling. Their relatively long half-life allows larger-scale radiosynthesis by a nuclear facility for delivery to regional hospitals. The initial study revealed that 3-[^76/77^Br]BAMT *in vivo* is metabolized over time and debrominated, resulting in slow blood clearance and high accumulation of free bromine in normal organs 100. In a subsequent study, ^76^Br or ^77^Br were radiolabeled onto L-α-methyl-phenylalanine, which lacks the phenolic hydroxyl group compared to L-α-methyl-tyrosine (**Figure 1**). The 2-[^76/77^Br]bromo-α-methyl-L-phenylalanine (2-[^76/77^Br]BAMP) demonstrated preferable *in vivo* stability and faster blood clearance than 3-[^76/77^Br]BAMT and even [^18^F]FAMT. In particular, 2-[^76/77^Br]BAMP displayed a much lower kidney accumulation at 60 min post-injection than 3-[^76/77^Br]BAMT and [^18^F]FAMT. Interestingly, the tumor-to-muscle uptake ratio of 2-[^76/77^Br]BAMP continued increasing from 1 to 3 h post-injection due to quick muscle clearance 101.

The ideal characteristic of 2-[^76/77^Br]BAMP subsequently reignited the idea of ^18^F radiolabeling and drove the development of [^18^F]fluoro-α-methyl-L-phenylalanine ([^18^F]FAMP, **Figure 1**). Among regioisomer and stereoisomer of α-methyl-phenylalanine tested, 2-[^18^F]fluoro-α-methyl-L-phenylalanine ([^18^F]FAMP) featured high tumor uptake via LAT1 (higher than [^18^F]FAMT) and low kidney accumulation (significantly less than [^18^F]FAMT). Similar to the 2-[^76/77^Br]BAMP tumor uptake pattern, [^18^F]FAMP clearly visualized orthotopic bladder tumors at 3 h post-injection because of quick muscle clearance 102. Fascinatingly, the *in vivo* stability, pharmacokinetics, and LAT1 tumor uptake of 2-α-methyl-L-phenylalanine analog remains unaffected when α-particle emitter astatine-211 (^211^At) is integrated to construct the therapeutic analog 2-[^211^At]-astato-α-methyl-L-phenylalanine ([^211^At]AAMP) 103. Again, similar to [^18^F]FAMT, probenecid loading also reduces [^211^At]AAMP kidney uptake and enhances tumor accumulation, improving its therapeutic effect in murine tumor models 104.

The direct electrophilic substitution synthesis route of [^18^F]FAMP produces low radiochemical yield (20-30%) and specific activity (2-3 MBq/*μ*mol) 105. However, a proposed late-stage fluorination method using *N*-pivaloyl chloride-protected (mesityl)(aryl)iodonium salt precursor could obtain [^18^F]FAMP within 120 min (radiochemical yield 21.4%, radiochemical purity >95%, and specific activity >37 GBq/*μ*mol) 86. Thus, a large-scale clinical trial is awaited to pave the way for [^18^F]FAMP clinical use.

### 3. [^18^F]FIMP

Another research group in Japan developed an α-methyl-L-phenylalanine analog based on screening using human LAT1 and LAT2 overexpressed cell lines. They found that *(S)*-2-amino-3-[3-(2-[^18^F]-fluoroethoxy)-4-iodophenyl]-2-methylpropanoic acid ([^18^F]FIMP, **Figure 1**) was among the LAT1 high-affinity substrate (IC_50_ 88.5 ± 13.5 *μ*M). The ^18^F radiolabeling via nucleophilic substitution method produced [^18^F]FIMP with >98% radiochemical purity and high specific activity up to 122 GBq/*μ*mol 106.

In a study of animal tumor and inflammation model, [^18^F]FIMP was not metabolized into protein; thus, [^18^F]FIMP was stable and excreted almost intact in urine. [^18^F]FIMP has similar tumor uptake to [^18^F]FDG, while uptake in inflamed lesions was significantly less. However, [^18^F]FIMP muscle uptake was higher than [^18^F]FDG; thus, the [^18^F]FIMP tumor-to-muscle ratio was less than [^18^F]FDG. Compared to [^18^F]FET, inflammation uptake of [^18^F]FIMP was higher. Nevertheless, since [^18^F]FIMP has higher tumor uptake and less muscle uptake than [^18^F]FET, [^18^F]FIMP PET image has significantly better contrast 106. A further investigation detailing [^18^F]FIMP specificity toward other amino acid transporters revealed that [^18^F]FIMP is also a substrate of ATB^0,+^ (SLC6A14), just like [^18^F]FET 107. However, ATB^0,+^ (SLC6A14) protein expression is too low to be detected in healthy tissues 108. Thus, the extended muscle uptake of [^18^F]FIMP is likely due to its pharmacokinetics rather than transport by ATB^0,+^. The ATB^0,+^ is significantly increased in some cancers (pancreatic, cervical, colon, and estrogen-positive breast cancers); thus [^18^F]FIMP perhaps has potential for cancer detection via ATB^0,+^-targeting. However, this subtopic is beyond the scope of the current review.

The first-in-human investigation of [^18^F]FIMP PET has been done recently in healthy volunteers and brain tumor patients in comparison with [^11^C]MET and [^18^F]FDG. [^18^F]FIMP rapidly cleared from the blood, resulting in very low background activity 1-hour post-injection. As expected, a clear margin of glioblastoma (grade IV glioma) can be demonstrated with higher contrast than that with [^11^C]MET and [^18^F]FDG 109. Due to their low brain physiological uptake, amino acid-based PET radiotracers have long been expected to replace [^18^F]FDG in various scenarios in neurooncology cases 27, 110. One of their fundamental problems is their affinity toward LAT2. LAT2 is expressed on the plasma membrane of neuronal axons in the cerebral cortex, choroid plexus, subfornical organ, and hypothalamus 111. In inflammation-related neurooncology cases like radiation necrosis, amino acid PET radiotracer should also be able to discriminate cancer lesions from inflamed tissues. Current evidence favors amino acid PET radiotracers ([^11^C]MET, [^18^F]FET, and [^18^F]DOPA) for post-radiotherapy cases; however, they are inconclusive in terms of which PET radiotracers are the best 112-114. Thus, it is interesting to watch the diagnostic performance of [^18^F]FIMP PET in neurooncology cases against the established PET radiotracer [^18^F]FET 5, especially considering that [^18^F]FIMP is less prone to post-radiotherapy inflammation 115.

### 4. [^18^F]FELP

The [^18^F]FET (*O*-[^18^F]fluoroethyl-L-tyrosine) is arguably one of the most popular amino acid-based PET radiotracer. Even though [^18^F]FET is not LAT1-specific, [^18^F]FET has an efficient radiosynthesis route, allowing a large quantity of production for routine clinical use and distribution to surrounding hospitals 5. Inspired by [^18^F]FET, a *para*-fluoroethyl of alkylated phenylalanine, *p*-(2-[^18^F]fluoroethyl)-L-phenylalanine (*(S)*-4-[^18^F]FELP) was successfully synthesized (>95% radiochemical purity, up to 37% yield, and up to 69 GBq/*μ*mol specific activity within 90 min) and showed LAT1-selective uptake in animal tumor models 116. Recently, the *ortho*- and *meta*-substitution versions of fluoroethyl phenylalanine and their enantiomers were studied. Among these analogs, 2-[^18^F]-2-fluoroethyl-L-phenylalanine (*(S)*-2-[^18^F]FELP or [^18^F]FELP, **Figure 1**) was the most LAT1-specific (*K*_m_ 8.72―16.36 *μ*M, in competition with JPH203, a LAT1-specific and potent inhibitor).

**Figure 6:**
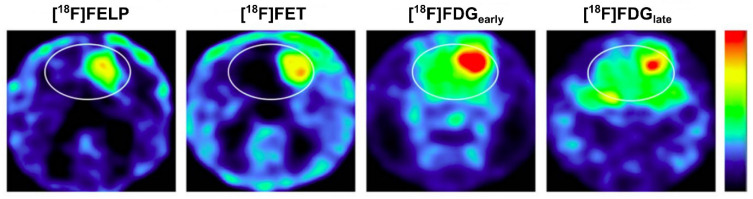
[^18^F]FELP depicted a tumor uptake level similar to [^18^F]FET in Micro PET images of orthotopic glioblastoma rat models, with less background uptake (both images were taken 100 min post-injection). A later [^18^F]FDG image (240 min post-injection) demonstrated less normal brain tissue uptake than the early image, but those uptakes remained higher than that on [^18^F]FELP and [^18^F]FET images. Adapted from 117. Copyright © 2019, The Author(s). Published under Creative Commons Attribution 4.0 License (CC-BY License, https://creativecommons.org/licenses/by/4.0/).

Imaging comparison between [^18^F]FELP and [^18^F]FET in an orthotopic glioblastoma rat model demonstrated comparable SUV and tumor-to-background ratios (**Figure 6**) 117. In a similar animal model, [^18^F]FELP also showed its potential to discriminate glioblastoma lesions from radiation necrosis, as well as [^18^F]FET 118. Until now, [^18^F]FELP PET studies in humans have not yet been reported.

### 5. LAT1 inhibitor-based PET radiotracers

A high-affinity, non-transportable LAT1-specific blocker has been successfully synthesized based on their structure-activity relationship 119. This LAT1 inhibitor, JPH203 (nanvuranlat; IC_50_ 0.14 *μ*M, no transport via LAT2) 120, underwent a series of *in vitro* and *in vivo* studies confirming its potential for a *pan-cancer* therapeutic agent 26. A Japanese pharma company conducted a phase III clinical trial of JPH203 and investigated several variants of LAT1 inhibitors. They also investigated NKO-028 and NKO-035, two amino acid derivative LAT1-selective substrates, as prospective PET radiotracers for solid tumors 121.

[^18^F]NKO-028 and [^18^F]NKO-035 were claimed to be LAT1-specific with high affinity. [^18^F]NKO-028 synthesis took 80 min with a 30% radiochemical yield, while automated synthesis could obtain [^18^F]NKO-035 with 1,000 GBq/*μ*mol specific activity and >99% radiochemical purity (50:50 enantiomeric mixture of L- and D-form) in 68 min 122. Both PET radiotracers have no specific accumulation in major organs. High [^18^F]NKO-028 accumulation in the spleen, kidney, and other organs was decreased rapidly after administration 123. On the other hand, when [^18^F]NKO-035 was tested in rat inflammation models, physiological uptake remained observed on the kidneys and pancreas at 60 min post-administration. Nevertheless, [^18^F]NKO-035 is relatively stable *in vivo* (90% unchanged at 75 min) and demonstrated low uptakes on inflammatory lesions 124.

A human dosimetry study confirmed that [^18^F]NKO-035 has minimal accumulation in normal tissues except in the bladder, kidneys, and urinary tracts. A typical [^18^F]NKO-035 injection dose of ±221 MBq exposes each patient to an effective dose of 4.4 mSv (including a CT dose of 2.3 mSv) 125. For comparison, almost half of the effective dose in a whole-body [^18^F]FDG PET/CT scan (±244 MBq dose of [^18^F]FDG) generated 4.2 mSv effective dose, without CT dose included 126. Another human dosimetry study designed to calculate the 2 Gy equieffective dose (EQD2(α/β)) parameter using [^18^F]NKO-035 implies the possibility of developing NKO-035 as a LAT1-targeting α-particle emitter therapy using ^211^At. The dynamic PET acquisition of [^18^F]NKO-035 demonstrated very low background uptake at 60 min post-injection, potentially delivering images with higher contrast than [^18^F]FDG PET 127. It is very compelling to find out whether the slow kidney clearance of [^18^F]NKO-035 is also facilitated by a similar mechanism to [^18^F]FAMT. If so, a similar approach to inhibit OAT1 can be implemented. Clinical translation of [^18^F]NKO-035 is ongoing; thus, we are excited to see its diagnostic performance against [^18^F]FDG and other amino acid-based PET radiotracers in various cancers 128.

Several prospective LAT1 inhibitors have been shortlisted from a virtual screening of large databases based on dynamic pharmacophores generated from molecular dynamic simulations. In particular, two of the 13 compounds demonstrated a highly potent LAT1 inhibition in a competitive *in vitro* assay against L-histidine, the LAT1 natural amino acid substrate with the highest affinity 129. Developing these potent LAT1 inhibitors as PET radiotracers in future studies might be promising.

## Conclusions and Perspectives

LAT1 is a *pan-cancer* molecular target promising for cancer diagnosis. LAT1 overexpression in cancer is also prognostic for survival and, thus, may serve as a monitoring biomarker of therapeutic responses. Clinical evidence showed that LAT1 targeting PET imaging can discriminate malignant tissue from benign components in a heterogeneous tumor mass. The lack of LAT1 expression in non-cancer pathologies (e.g., benign tumors, inflammatory tissues, radiation necrosis) allows these PET radiotracers to delineate the actual borders of malignant tissue with high accuracy. Since the dynamics of LAT1 expression highly correlate with the behavior of cancer cells, LAT1-specific PET radiotracers would firmly stand on a wide spectrum of cancer management, from early detection, differential diagnosis, and therapy response monitoring to long-term prognostication. Therefore, LAT1-specific PET radiotracers may compensate for the limitations of the current standard oncologic PET radiotracer. Major advantages and known challenges of LAT1 targeting using PET radiotracers discussed in this review are summarized in **Table 2**.

As previously mentioned, most PET radiopharmaceuticals claimed to be LAT1-specific are LAT1 substrates, hence prone to washout due to LAT1 efflux. Even though the absolute tumor uptake of the current LAT1-specific PET radiotracers has a lower quantity than [^18^F]FDG, the lack of LAT1 expression in the plasma membrane of normal tissues allows tumor accumulation of these PET radiotracers to generate clinical PET images with a relatively high tumor-to-background (or T/N) ratio (see **Table S1**). In the future, the development of LAT1-specific PET radiotracers equipped with favorable characteristics such as high affinity, good biodistribution, early uptake, and faster blood clearance is warranted. A non-transportable LAT1 inhibitor may also be a favorable characteristic; however, tumor uptake can be much improved if a LAT1-specific PET radiotracer could be internalized into the cytoplasm and retained there without the possibility of being effluxed. The detailed structure of LAT1, including the binding pocket, gating mechanism, and substrate/inhibitor preferences, should be the basis for further development of LAT1-specific PET radiopharmaceuticals.

In conclusion, several LAT1-specific PET radiotracers are in the final stages of preclinical studies or even phase II/III clinical trials. Once these LAT1-specific PET radiotracers are approved for clinical use by the government regulatory bodies, we may expect improvement in multiple aspects of cancer management.

## Supplementary Material

Supplementary tables.

## Figures and Tables

**Table 1:** Amino acid PET radiotracers (reviewed in meta-analyses) and their respective transporters

Amino acid PET tracers	Amino acid transporter(s) responsible for uptake	Organs of high physiological uptake*	Cancer types
[^11^C]MET	LAT1-4, SNAT1, SNAT2, SNAT4, ASCT2 29, 30	Liver, pancreas (↓), salivary glands, lachrymal glands, bone marrow, bowels, kidney, and bladder 31	Brain 32 (glioma 33, high-grade glioma 34), parathyroid adenoma 35, multiple myeloma 36
[^18^F]FET	LAT2 (dominant), ATB^0,+^, System B^0^, LAT1 (poor) 30	Pancreas, kidneys, liver, heart, colon, and muscle 31	Brain (high-grade glioma 34, recurrent glioma 37, metastases 38)
[^18^F]FDOPA	LAT1 and LAT2 (similar affinity) 39	Urinary bladder, gallbladder, biliary tract, pancreas, kidney, basal ganglia, liver, and adrenal glands 40	Neuroendocrine tumors (multiple sites 41, intestinal neuroendocrine tumors 42, medullary thyroid cancer 43), glioma 44
[^18^F]FACBC	ASCT2, SNAT2, LAT1, LAT2 45	Liver, pancreas (↓), heart, kidneys, spleen, allbladder, bone marrow (↓), adrenal glands, muscle (↑), and stomach 46	Prostate (primary lesion and primary lymph node metastasis) 47, recurrent prostate cancer 48, high-grade glioma 49
[^18^F]FAMT	LAT1 29	Kidney and urinary bladder 50	Various cancer 51

***Notes:*** * Ranked from the highest, (↓)/(↑) decreased/increased uptake over time.

**Table 2:** LAT1-specific PET radiopharmaceuticals: Advantages and challenges

LAT1-specific PET radio-pharmaceuticals	Advantages as a LAT1-targeting radiotracer	Challenges
[^18^F]FBPA	Clinically used to predict boron accumulation in tumors before BNCT Suitable for malignant melanoma due to prolonged retention No uptake in inflammation and benign lesions. Typical T/N ratio for malignancy is >2.5 High contrast whole body image might be obtained as early as 20‒30 min p.i.	Limited availability (Japan, Finland, China, United States, Taiwan) Limited specific activity using the current radiosynthesis method (a new synthesis method is available) *In vivo* deboronation Poor water solubility (can be solved with the addition of fructose)
[^18^F]FBY	Potentially a better predictor for boron accumulation before BNCT Excellent stability against defluorination or deboronation T/N ratio ranges from 2.3 to 24.5 High contrast whole body image might be obtained as early as 30‒40 min p.i.	Limited availability (China) Need more clinical data
[^18^F]BBPA	Potentially a better predictor for boron accumulation before BNCT Can be obtained in Curie-level amount Excellent stability against defluorination or deboronation Mean T/N ratio in the brain = 18.7 High contrast whole body image might be obtained as early as 30‒40 min p.i.	Uptake was found in non-malignant lesions Limited availability (China) Need more clinical data
[^18^F]FAMT	Clinically proven as a diagnostic and prognostic tool No uptake in inflammation and benign lesions High contrast whole body image might be obtained as early as 40‒60 min p.i.	Limited availability (Japan) Limited specific activity using the current radiosynthesis method (a new synthesis method is available) High kidney retention (can be reduced with probenecid preloading)
[^18^F]FAMP	Similar to [^18^F]FAMT regarding normal tissue distribution, yet higher tumor uptake and lower kidney retention	Limited availability (Japan) Limited specific activity using the current radiosynthesis method (a new synthesis method is available) High pancreas uptake No clinical data is available yet
[^18^F]FIMP	High contrast whole body image might be obtained at around 60 min p.i. No uptake in radiation necrosis (inflammation)	Limited availability (Japan) Very limited clinical data
[^18^F]FELP	No uptake in radiation necrosis and inflammatory tissues	Limited availability (Belgium) No clinical data is available yet
[^18^F]NKO-035	A high contrast whole body image might be obtained at around 60 min p.i. Low uptake in inflammatory tissue	Limited availability (Japan) Relatively high kidney retention Very limited clinical data

***Note:* p.i.** post injection

## Abbreviations

ASCT2: Alanine, Serine, Cysteine Transporter 2
BNCT: boron neutron capture therapy
[^76/77^Br]BAMP: 2-[^76/77^Br]bromo-α-methyl-L-phenylalanine
[^76/77^Br]BAMT: 3-[^76/77^Br]bromo-α-methyl-L-tyrosine
[^11^C]MET: L-[*methy*l-^11^C]methionine or 2-amino-4-[^11^C]methylsulfanyl-butanoic acid
[^18^F]FAMP: 2-[^18^F]fluoro-α-methyl-L-phenylalanine
[^18^F]FAMT: 3-[^18^F]fluoro-α-methyl-L-tyrosine
[^18^F]FBPA: 4-borono-2-[^18^F]fluoro-D,L-phenylalanine
[^18^F]FBY: [^18^F]fluoroboronotyrosine
[^18^F]FDG: [^18^F]fluorodeoxyglucose or 2-deoxy-2-[^18^F]fluoro-D-glucose
[^18^F]FDOPA: [^18^F]fluorodopa or 3,4-dihydroxy-6-[^18^F]fluoro-L-phenylalanine
[^18^F]FELP: 2-[^18^F]-2-fluoroethyl-L-phenylalanine
[^18^F]FET: *O*-[^18^F]fluoroethyl-L-tyrosine
[^18^F]FIMP: *(S)*-2-amino-3-[3-(2-[^18^F]-fluoroethoxy)-4-iodophenyl]-2-methylpropanoic acid
[^123^I]IMT: 3-[^123^I]iodo-α-methyl-L-tyrosine
LAT1: L-type amino acid transporter 1
MIP: mean intensity projection
PET: positron emission tomography
SUV_max_: maximum standardized uptake volume
SUV_peak_: peak standardized uptake volume
