# Supplementary material for: LAT1-specific PET radiotracers: Development and clinical experiences of a new class of cancer-specific radiopharmaceuticals
Source: Theranostics. 2025 Jan 2;15(5):1864–78. doi: 10.7150/thno.99490 (PMC11780518; doi:10.7150/thno.99490)
Supplement: Supplementary file 1 — Supplementary tables. [file thnov15p1864s1.pdf]

Supplementary Table S1. Amino acid transporter overexpression in epidemiologically relevant cancers (based on global cancer burden data from GLOBOCAN 2022)

**Incidence rank** (%)	**Mortality rank** (%)	**Cancer site**	**LAT1**	**ASCT2**	**xCT**	**CAT (1/2/3)**	**SNAT2**
**1** (12.4)	**1** (18.7)	Lung	✓	✓	✓		
**2** (11.6)	**4** (6.9)	Female breast	✓	✓	✓	✓	✓
**3** (9.6)	**2** (9.3)	Colorectum	✓	✓	✓	✓[1,2]	
**4** (7.3)	**8** (4.1)	Prostate	✓	✓	✓		
**5** (4.9)	**5** (6.8)	Stomach	✓	✓		✓[1]	
**6** (4.3)	**3** (7.8)	Liver	✓	✓	✓	✓[1]	
**7** (4.1)	**24** (0.5)	Thyroid	✓		✓	✓[3]	
**8** (3.3)	**9** (3.6)	Cervix uteri	✓	✓			✓
**9** (3.1)	**13** (2.3)	Bladder	✓				
**10** (2.8)	**11** (2.6)	Non-Hodgkin lymphoma	✓	✓	✓		
**11** (2.6)	**7** (4.6)	Esophagus	✓	✓	✓		
**12** (2.6)	**6** (4.8)	Pancreas	✓		✓		✓
**13** (2.4)	**10** (3.1)	Leukemia	✓			✓[1,2]	
**14** (2.2)	**16** (1.6)	Kidney	✓	✓			
**15** (2.1)	**19** (1.0)	Corpus uteri*	✓	✓			
**16** (2.0)	**15** (1.9)	Lip, Oral cavity	✓	✓	✓		
		Tongue cancer	✓		✓		
**17** (1.7)	**22** (0.6)	Melanoma	✓		✓	✓[1]	
**18** (1.6)	**14** (2.1)	Ovarium	✓	✓		✓[3]	✓
**19** (1.6)	**12** (2.6)	Brain, CNS	✓				
		Neuroblastoma	✓	✓			
		Glioma, glioblastoma	✓		✓		
**20** (0.9)	**18** (1.1)	Larynx	✓		✓		
**21** (0.6)	**17** (1.2)	Multiple myeloma	✓	✓			

Note: *including endometrial cancer; **1** CAT1, **2** CAT2, **3** CAT3.
Summarized from Scalise (2020)[1], Hafliger (2019)[2], Zhang (2020)[3], and Bray (2024)[4].

References

1. Scalise, M.; Console, L.; Rovella, F.; Galluccio, M.; Pochini, L.; Indiveri, C. Membrane transporters for amino acids as players of cancer metabolic rewiring. *Cells* **2020**, *9*, 2028, doi: 10.3390/cells9092028.
2. Hafliger, P.; Charles, R.P. The L-Type Amino Acid Transporter LAT1-An Emerging Target in Cancer. *Int J Mol Sci* **2019**, *20*, doi: 10.3390/ijms20102428.
3. Zhang, J.; Xu, Y.; Li, D.; Fu, L.; Zhang, X.; Bao, Y.; Zheng, L. Review of the Correlation of LAT1 With Diseases: Mechanism and Treatment. *Front Chem* **2020**, *8*, 564809, doi: 10.3389/fchem.2020.564809.

4. Bray, F.; Laversanne, M.; Sung, H.; Ferlay, J.; Siegel, R.L.; Soerjomataram, I.; Jemal, A. Global cancer statistics 2022: GLOBOCAN estimates of incidence and mortality worldwide for 36 cancers in 185 countries. *CA: A Cancer Journal for Clinicians* **2024**, *74*, 229-263, doi: https://doi.org/10.3322/caac.21834.

Supplementary Table S2. LAT1-specific PET radiopharmaceuticals in clinical studies (in comparison with [^{18}F]FDG)

Radiopharmaceutical, Study (year)	*N*	Tumor & other pathology examined (*n* of patient)	Number of malignant and/or benign lesions	Study Design	Advantageous feature(s) in comparison with [^{18}F]FDG in the study
**[^{18}F]FAMT**					
Inoue (1999) [1]	20	Brain tumors	23 mlgn	Pro	Provide high-contrast images of brain tumors
Watanabe (2000) [2]	74	Musculoskeletal tumors: 24 bone, 48 soft tissue	22 mlgn, 53 bngn	Pro	Superior for differentiation between benign and malignant tumors. SUV correlates with tumor grade
Inoue (2001) [3]	19	Lung canc (10), mlgn myeloma (2), chondrosarcoma (1), prostate canc (1), mlgn lymphoma (1), mlgn of unknown origin (1), Schwannoma (1), sarcoidosis (2)	57 mlgn	Pro	Clinically useful in the diagnosis of malignancy. Effective depiction of primary and metastasis lesions in cardiac region or the brain
Ahmed (2001) [4]	22	Schwannoma	25 bngn	Pro	The most reliable for differentiation of schwannoma from malignancy (compared with CT, MRI & ^{18}F-FDG)
Hatayama (2003) [5]	16	Hemangioma	16 bngn	Pro	SUV correlates well with lesion size while that of ^{18}F-FDG was not
Sato (2003) [6]	14	Gliomatosis cerebri (8): Anaplastic (1), grade II astrocytoma (4) grade III astrocytoma (3). Non-neoplastic disease (6)	8 mlgn, 6 bngn	Pro	Increased uptake strongly suggests malignancy. Only one false-negative result due to high intracranial pressure (diffuse tumor infiltrating the whole brain). All true negative results are in the non-neoplasm group.
Suzuki (2004) [7]	52	Tumors on shoulder girdle	14 mlgn, 38 bngn	Pro	Similarly limited for malignancy detection
Suzuki (2005) [8]	57	Fatty tissue tumor (57): liposarcoma (25), lipoma (32)	25 mlgn, 32 bngn	Pro	Equally accurate to differentiate lipoma from liposarcoma (except well-differentiated type)
Higuchi (2006) [9]	3	Pancreatic lesions (3): SQC (1), pancreatitis (2)	1 mlgn, 2 bngn	Pro	Demonstrated true positive for ca and true negative for pancreatitis
Kaira (2007) [10]	41	Lung canc (17): AC (9), SQC (6), NSCLC (2). Sarcoidosis (24)	17 primary mlgn, 16 LNM mlgn, 24 bngn	Pro	More efficient to distinguish sarcoidosis from malignancy
Kaira (2007) [11]	50	NSCLC: AC (28), SQC (18), non-AC (22)	68 mlgn	Pro	More specific for lymph node staging, similarly sensitive for primary tumor
Miyakubo (2007) [12]	43	Maxillofacial tumors: SQC (34), rhabdomyosarcoma (1), mucoepidermoid ca (1), LNM (14). Benign lesions (7)	36 primary mlgn, 14 LNM mlgn, 7 bngn	Pro	Similar accuracy but higher contrast (by visual assessment)
Kaira (2009) [13]	43	Thoracic tumors (35): SQC (9), AC (19), bronchoalveolar ca (3), atypical SQC (2), LCC (1), carcinoid (1)	35 mlgn, 6 bngn	Pro	Higher specificity and slightly lower sensitivity. Closely correlated with angiogenesis (VEGF, CD31, CD34) and cell proliferation (Ki-67)
Kaira (2009) [14]	37	NSCLC (37): AC (18), SQC (15), LCC (2), others (2)	37 mlgn	Ret	More correlated with angiogenesis (VEGF) and proliferative activity (Ki-67)
Kaira (2009) [15]	98	NSCLC (98): AC (57), SQC (31), LCC (5), others (5)	98 mlgn	Ret	Independent and stronger predictor of OS for AC. More accurate for DFS rate prediction
Nakano (2009) [16]	14	Pancreatic lesions (13): pancreatic ca (7), pancreatitis (6)	7 mlgn, 6 bngn	Pro	Less uptake in benign pancreatic lesions
Kaira (2010) [17]	18	Lung canc (18): AC (10), SQC (4), LCC (1), SCLC (3)	18 mlgn	Pro	Uptake after treatment can predict prognosis
Kaira (2010) [18]	59	NSCLC (59): AC (31), SQC (20), LCC (2), others (6)	59 mlgn	Pro	Primary tumor uptake compared with LAT1 expression: Prognostic factor (univariate analysis). Less powerful prognostic factor for OS (multivariate analysis)
Miyashita (2010) [19]	25	Oral SQC	21 mlgn	Pro	Lower sensitivity but better correlation with cell proliferation (Ki-67)
Oriuchi (2010) [20]	88	NSCLC (88): AC (57), SQC (31)	88 mlgn	Pro	Potential prognostic marker for pulmonary AC.
Sohda (2010) [21]	21	Esophageal SQC	21 primary mlgn, 33 LNM mlgn	Pro	Higher specificity but lower sensitivity than ^{18}F-FDG and CT
Tian (2011) [22]	36	Musculoskeletal tumors	13 mlgn, 23 bngn	Pro	Compared with ^{11}C-Choline and ^{18}F-FDG: Lowest sensitivity but highest specificity
Isoda (2012) [23]	11	Multiple myeloma	34 mlgn bone lesions	Pro	Lower lesion-to-background ratio, similar lesion-to-mediastinum ratio
Morita (2012) [24]	21	Bone mets: lung ori (9), esophagus ori (6), stomach ori (1), bile duct ori (1), pancreas ori (1), thymoma ori (1), prostate ori (2)	72 mlgn	Pro	Detect bone metastasis lesions regardless the bone lesion type
Kim (2013) [25]	27	Oral SQC	27 mlgn (20 bone marrow invasion)	Pro	Accurate tumor delineation/tumor volume prediction
Nobusawa (2013) [26]	68	Oral SQC	68 primary mlgn, 279 LNM mlgn	Ret	Higher specificity for LNM, similar specificity for primary tumors
Sohda (2014) [27]	30	LNM of esophageal SQC	30 LNM mlgn	Ret	LNM uptake is a good predictor of complete response to chemoradiotherapy
Sohda (2014) [28]	40	Esophageal SQC	40 mlgn	Ret	Primary tumor uptake is a good predictor of complete response after chemoradiotherapy
Sohda (2014) [29]	42	Esophageal SQC	42 mlgn	Ret	Primary tumor uptake is a good predictor of DFS in operable tumors
Suzuki (2014) [30]	42	Esophageal SQC	42 mlgn	Ret	Uptake correlated wih cell proliferation (Ki-67) and angiogenesis (CD34)

Supplementary Table S2. LAT1-specific PET radiopharmaceuticals in clinical studies (in comparison with [^{18}F]FDG)

Radiopharmaceutical, Study (year)	*N*	Tumor & other pathology examined (*n* of patient)	Number of malignant and/or benign lesions	Study Design	Advantageous feature(s) in comparison with [^{18}F]FDG in the study
Kim (2015) [31]	25	Oral SQC	25 mlgn	Ret	More accurate tumor volume prediction. Not overestimated by inflammations
Kodaira (2016) [32]	24	Liver mets (59): AC (21), SQC (23), NE tumor ori (9), carcinoid (6)	59 mlgn	Ret	Could detect metastatic liver lesions from various types of primary tumors (pancreas, lung, rectum, esophagus, and oral canc), except NE tumor
Kaira (2016) [33]	95	Advanced lung canc, stage IIIA (18), stage IIIB (23), stage IV (54): AC (15), SQC (21), LCC (5), SCLC (8), other (6)	55 mlgn	Pro	Metabolic response was an independent factor to predict the OS after first-line chemotherapy
Horiguchi (2017) [34]	38	Glioma: WHO stage II (12), stage III (12), stage IV (14)	12 low grade glioma, 26 high grade	Ret	Higher T/N ratio, able to differentiate low- and high-grade gliomas
Honjo (2017) [35]	142	Esophageal SQC	142 mlgn	Ret	Uptake (and LAT1 expression) correlates with metastasis-associated gene 1 (MTA-1)
Sohda (2018) [36]	82	Esophageal SQC	82 mlgn	Pro	Lower rate of nonspecific uptake
Sohda (2018) [37]	6	Locally advanced esophageal SQC (without distant mets)	6 mlgn	Pro	More accurate modality for complete response judgment at 1 month after chemoradiotherapy
Kumasaka (2018) [38]	112	NSCLC (112): AC (72), SQC (28), other (12)	112 mlgn	Pro	Pretreatment MTV was highly prognostic of OS (median follow up: 1.58 years)
Kim (2019) [39]	160	Oral SQC	160 mlgn	Ret	MTV predict OS (mean follow up: 4.5 years)
Kuriyama (2020) [40]	41	Esophageal SQC	41 mlgn	Ret	High SUV_{max} was a predictor of hot tumor immune status (high PD-L1, high $CD8^{+}$ lymphocytes within tumors)
Shimizu (2020) [41]	75	NSCLC (75): AC (51), non-AC (24)	75 mlgn	Ret	High uptake was associated with staging, initial treatment, and tumor immune marker (PD-L1) expression. No significant correlations between tumor-infiltrating lymphocytes and SUV_{max}
Suzuki (2021) [42]	31	Intraosseus ca of oral canc (18), maxillo-mandibular actinomycosis/ actinomycotic osteomyelitis (13)	18 mlgn, 13 bngn	Ret	Negative uptake in progressive actinomycotic osteomyelitis lesions (bone resorption with irregular margins, very similar to intraosseus carcinoma) clearly differentiate these lesions from malignancies
**[^{18}F]FBPA**					
Yoshino (1997) [43]	5	Brain mets (5): breast origin (2), lung origin (3)	8 mlgn	Pro	Along with [^{18}F]FDG, useful to differentiate between tumor progression and radiation necrosis
Mishima (1997) [44]	4	Melanoma (3), brain mets (rhabdomyosarcoma) (1)	6 mlgn	Pro	(※) T/N ratio ranged from 2.3 to 3.38
Kabalka (1997) [45]	2	Glioblastoma multiforme (2)	2 mlgn	Pro	(※) Can be used to determine the distribution of brain tumor tissue and estimate radiodosimetry before BNCT
Imahori (1998) [46]	33	Primary glioma (33): glioblastoma multiforme (13), astrocytoma WHO grade II (8), and anaplastic astrocytoma WHO grade III (12)	25 high grade glioma, 8 low grade glioma	Pro	(※) T/N ratio ranged for high grade glioma were 1.55 – 5.15, while for low grade glioma were 0.8 – 1.95; hence suggested cut off T/N ratio for malignancy was >2
Takahashi (2003) [47]	22	Glioblastoma (9), anaplastic astrocytoma (7), diffuse astrocytoma (6)	16 high grade glioma, 6 low grade glioma	Pro	(※) The mean survival time was significantly shorter in patients with high uptakes
Havu-Aurén (2007) [48]	10	Sporadic & neurofibromatosis (NF2)-related intracranial tumors (10): meningioma (4), Schwannoma (6)	10 bngn	Pro	(※) SUV_{max} (2.4 – 7.6) was two- to fourfold higher in tumor as compared with normal brain and independent of NF2 status
Miyatake (2007) [49]	6	Malignant meningioma (6): papillary (2), atypical (1), anaplastic (1), sarcoma transformed from a meningioma (1)	10 mlgn	Pro	(※) T/N ratio ranged from 2 to 5
Ariyoshi (2007) [50]	4	Recurrent oral canc & cervical LMN (4): SQC (2), AC (1), mucoepidermoid ca (1)	4 mlgn	Pro	(※) T/N ratios ranged from 2.2 to 4.0
Kankaanranta (2007) [51]	12	Head & neck canc (12): SQC (10), adenocystic carcinoma (1), transitional cell ca (1)	12 mlgn	Pro	(※) T/N ratios ranged from 2.5 to 6.3 (median, 3.6)
Fuwa (2008) [52]	5	Head & neck canc (5): SQC (4), adenoid cystic canc (1)	5 mlgn	Pro	(※) T/N ratios ranged from 4.0 to 12.5
Nairai (2009) [53]	12	Glioblastoma multiforme (11), anaplastic astrocytoma (1)	12 mlgn	Pro	(※) High linear correlation between T/N ratio and boron concentration (r^2=0.89), similar to [^{11}C]MET
Kimura (2009) [54]	6	Recurrent oral canc & cervical LMN (6): SQC (2), AC (2), melanoma (1), mucoepidermoid ca (1)	6 mlgn	Pro	(※) T/N ratios ranged from 1.9 to 4.0
Kankaanranta (2012) [55]	15	Head & neck canc (15), mostly SQC (Note: only 15 of 30 pts underwent [^{18}F]FBPA PET studies)	15 mlgn	Pro	(※)T/N ratios ranged from 2.5 to 9.0 (median, 4.1)
Kawabata (2013) [56]	19	High grade meningioma (19): papillary (2), atypical (4), anaplastic (11), sarcoma (1), rhabdoid (1)	19 mlgn	Pro	(※)T/N ratios ranged from 2.0 to 5.0 (18 pts have T/N ratio ≥ 2.7)

Supplementary Table S2. LAT1-specific PET radiopharmaceuticals in clinical studies (in comparison with [^{18}F]FDG)

Radiopharmaceutical, Study (year)	*N*	Tumor & other pathology examined (*n* of patient)	Number of malignant and/or benign lesions	Study Design	Advantageous feature(s) in comparison with [^{18}F]FDG in the study
Tani (2014) [57]	20	Head & neck canc (20): SQC (9), adenoid cystic ca (5), AC (1), basal cell ca (1), NE cell ca (1), salivary duct ca (1), pleomorphic adenoma (1), esthesioneuroblastoma (1)	20 mlgn	Pro	T/N ratios ranged from 1.35 to 5.98. Uptake was correlated with [^{18}F]FDG uptake (r^2=0.72).<br>[^{18}F]FDG SUV_{max} of ≥ 5 can predict [18F]FBPA SUV_{max} of ≥ 2.5.
Kobayashi (2016) [58]	10	Head & neck canc (10): tongue ca (2), oropharynx ca (1), adenoid cystic ca (1), paranasal ca (1), chondrosarcoma (1), buccal mucosa ca (1), soft palate ca (1), melanoma (1), nasopharyngeal ca (1)	10 mlgn	Pro	T/N ratios ranged from 1.73 to 5.21. In 9/10 patients, the spatial distribution of SUVs in the tumor volume positively correlates with that of [^{18}F]FDG. A tumor sub-volume with a high [^{18}F]FDG uptake may predict high accumulation of [^{18}F]FBPA
Watanabe (2017) [59]	7	Head & neck canc (7): SQC (2), adenoid cystic ca (2), rhabdomyosarcoma (2), melanoma (1)	7 mlgn	Pro	(※) T/N ratios ranged from 1.3 to 3.9.<br>SUV_{max} was correlated with that of [^{11}C]MET (r^2=0.72), but not significant
Morita (2018) [60]	28	Head & neck canc SQC (20), malignant melanoma (8)	28 mlgn	Pro	(※) The dynamics of mean T/N ratio in 30, 60, and 12 min of SQC (n = 20) were 3.21, 3.28, and 2.79; while mean T/N ratio of malignant melanoma (n = 8) were 7.89, 7.69, and 6.68.
Beshr (2018) [61]	12	Brain lesions (12): tumor recurrence [6: astrocytoma grade 2 (1), glioblastoma multiforme (1), oligo-astrocytoma grade 2 (1), oligo-astrocytoma grade 3 (1), anaplastic oligo-astrocytoma grade 3 (1), and hemangiopericytoma (1)] and necrosis [6: glioblastoma multiforme (4), meningioma (1), and mets of lung (1)].	12 mlgn	Pro	(※) PET parameters (SUV_{mean}, SUV_{max}, MTV, and total lesion uptake) of tumor recurrence (n=6) were significantly larger (2.9 vs 1.2; 4.6 vs 1.9; 44.9 vs 10.6; and 121 vs 12.3) than that in radiation necrosis (n=6)
Igaki (2020) [62]	120	Analysis 1 & 2: Head & neck SQC (11 & 27), AC (7 & 23), adult type sarcoma (5 & 13), juvenile type sarcoma (3 & 9), malignant melanoma (2 & 7), glioma (2 & 2), unknown histopathology (0 & 1)	120 mlgn	Pro	Analysis 1 (n = 30): SUV_{max} and T/N ratio correlated with that of [^{18}F]FDG (r^2=0.74 and r^2=0.65)<br>Analysis 2 (n = 82): SUV_{max} correlated with that of [^{18}F]FDG (r^2=0.48).<br>No convincible conclusions of clear correlations of SUVmax between [^{18}F]FDG and [^{18}F]FBPA
Lo (2020) [63]	25	Glioblastoma multiforme (12), anaplastic astrocytoma (4), diffuse intrinsic pontine glioma (1), meningioma (1), gingival canc (1), maxillary sinus canc (1), liposarcoma (1), tongue canc (1), buccal canc (1), thyroid canc (1), nasopharyngeal canc (1)	25 mlgn	Pro	(※) T/N ratios of glioblastoma multiforme ranged from 2.44 to 5.56, while other tumors ranged from 2.15 (nasopharyngeal ca) to 5 (buccal canc)
Isohashi (2022) [64]	82	Oral canc (13), soft tissue sarcoma (12), pharyngeal canc (10), salivary gland canc (6), external auditory canal canc (4), diffuse large B-cell lymphoma (3), laryngeal canc (3), skin canc (3), endometrial canc (3), cervical canc (3), mantle cell lymphoma (2), lung AC (2), breast canc (1), esophageal canc (2), ovarian canc (1), olfactory neuroblastoma (1), chronic lymphocytic leukemia (1), gallbladder canc (1), pancreatic canc (1), maxillary canc (1), bile duct canc (1), acute myeloid leukemia (1), canc of unknown primary (1), cervical LNM (1), Paget's disease (1).<br>Benign lesions [11**:** inflammatory granulation (2), abscess (2), radiation osteomyelitis (2), inflammatory lymphadenopathy (1), chemoradiotherapy post-treatment change (1), tonsillitis (1), cholecystitis (1), graft versus host disease (1)]	77 mlgn (22 primary/recurrent and 55 mets), 11 bngn	Pro	The SUV_{max} of [^{18}F]FDG PET/CT did not significantly differentiate malignant tumors from benign lesions, whereas SUV_{max} of [^{18}F]FBPA PET/CT was significantly higher for malignant tumors
Minoji (2023) [65]	3	Colorectal canc (3, all with T2N0M0)	3 mlgn	Pro	The SUV_{max} (compared to that of [^{18}F]FDG) for each tumor were 3.7 vs 17.44; 3.6 vs 8.48; and 3.98 vs 14.35.<br>All tumor lesions were scored 2 (of 4-point scales) in immunohistochemical analysis.
Watabe (2023) [66]	55	Primary lung canc [21: AC (13), SQC (5), NE ca (2), LCC (1)];<br>Lung mets [6: bladder NE ca (1), rectal ori (2), pharyngeal ori (1), breast ori (1)];<br>Thymoma [10: type AB (2), B1 (2), B2 (3), B3 (2), and atypical A (1)]; thymic canc (2), carcinoid (2), mesothelioma (1); and benign lesions (13)	42 mlgn, 13 bngn	Pro	SUV_{max} was positively correlated with LAT1 expression (*r*=0.61).<br>Benign lesions showed significantly lower SUV_{max} than those in maligancies.<br>SUV_{max} weakly correlates with that of [^{18}F]FDG (0.438, *P*=0.012)<br>Significantly lower uptake in the benign lesions, even when [^{18}F]FDG PET showed high uptake.<br>Thymomas, thymic canc, and mesotheliomas showed moderate uptake, while carcinoid showed low uptake.
**[^{18}F]FBY**					
Li (2021) [67]	18	Suspect of glioma (13) and normal volunteer (6)	14 glioma		(※) Elevated uptake is consistent with tumor grade and correlated with LAT1 expression (r^2 = 0.80, $P < 0.001$)<br>T/N ratio was 24.5 in high grade glioma and 2.3 in low grade glioma.

Supplementary Table S2. LAT1-specific PET radiopharmaceuticals in clinical studies (in comparison with [^{18}F]FDG)

Radiopharmaceutical, Study (year)	*N*	Tumor & other pathology examined (*n* of patient)	Number of malignant and/or benign lesions	Study Design	Advantageous feature(s) in comparison with [^{18}F]FDG in the study
Kong (2022) [68]	35	Primary diffuse glioma (12): WHO grade II (7), III (2), IV (3); Recurrent diffuse glioma (14): WHO grade II (2), III (6), IV (6); Brain mets (5): breast ori (2), lung ori (2), colorectal ori (1); Others (5): anaplastic pleomorphic xanthoastrocytoma (1), atypical meningioma (1), ganglioglioma (1), gliosis (1), and stable disease	9 low grade glioma, 17 high grade glioma, 7 other mlgn, and 2 bngn (gliosis & stable disease)	Pro	(※) Elevated uptake in most malignant brain tumors (SUV_{max} up to 3.0), while SUV_{max} of normal brain was 0.12. Significant T/N ratio was observed in primary GBM (22.6 ± 6.6), recurrent glioma (23.7 ± 8.3), and metastatic brain tumor (20.2 ± 3.8); while primary lower grade glioma displayed moderate T/N ratio (2.2 ± 1.3).
Kong (2022) [69]	23	Diffuse brain tumors (16): primary WHO grade IV (3), recurrent WHO grade II (1), grade III (6), and grade IV (6); Circumscibed brain tumor (7): mets of breast (2), mets of lung (2), mets of colorectal (1), anaplastic pleomorphic xanthoastrocytoma (1), and atypical meningioma (1)	23 mlgn	Pro	(※) MTVs obtained were larger than tumor volume obtained from contrast-enhanced area in MRI images especially in diffuse tumor; suggesting as a better delineation tool.

Abbreviations: AC adenocarcinoma, **bngn** benign, **ca** carcinoma, **canc** cancer, **DFS** Disease-free survival, **LCC** large cell carcinoma, **LNM** lymph node metastases, **mets** metastasis, **mlgn** malignant, **MTV** metabolic tumor volume, ***N*** number of patients, **NE** neuroendocrine, **NSCLC** non-small cell lung cancer, **ori** origin, **OS** overall survival, **Pro** prospective, **Ret** retrospective, **SCLC** small cell lung cancer, **SQC** squamous cell carcinoma, **SUV** standardized uptake value, **T/N** tumor-to-normal.
(※) [^{18}F]FDG was not used for comparison.

Supplementary Table S2. LAT1-specific PET radiopharmaceuticals in clinical studies (in comparison with [^{18}F]FDG)

REFERENCES

1. Inoue, T.; Shibasaki, T.; Oriuchi, N.; Aoyagi, K.; Tomiyoshi, K.; Amano, S.; Mikuni, M.; Ida, I.; Aoki, J.; Endo, K. ^{18}F alpha-methyl tyrosine PET studies in patients with brain tumors. *J Nucl Med* **1999**, *40*, 399-405.
2. Watanabe, H.; Inoue, T.; Shinozaki, T.; Yanagawa, T.; Ahmed, A.R.; Tomiyoshi, K.; Oriuchi, N.; Tokunaga, M.; Aoki, J.; Endo, K., et al. PET imaging of musculoskeletal tumours with fluorine-18 α-methyltyrosine: comparison with fluorine-18 fluorodeoxyglucose PET. *Eur J Nucl Med Mol Imaging* **2000**, *27*, 1509-1517, doi: 10.1007/s002590000344.
3. Inoue, T.; Koyama, K.; Oriuchi, N.; Alyafei, S.; Yuan, Z.; Suzuki, H.; Takeuchi, K.; Tomaru, Y.; Tomiyoshi, K.; Aoki, J., et al. Detection of malignant tumors: whole-body PET with fluorine-18 alpha-methyl tyrosine versus FDG: Preliminary study. *Radiology* **2001**, *220*, 54-62, doi: 10.1148/radiology.220.1.r01jl1654.
4. Ahmed, A.R.; Watanabe, H.; Aoki, J.; Shinozaki, T.; Takagishi, K. Schwannoma of the extremities: The role of PET in preoperative planning. *Eur J Nucl Med* **2001**, *28*, 1541-1551, doi: 10.1007/s002590100584.
5. Hatayama, K.; Watanabe, H.; Ahmed, A.R.; Yanagawa, T.; Shinozaki, T.; Oriuchi, N.; Aoki, J.; Takeuchi, K.; Endo, K.; Takagishi, K. Evaluation of hemangioma by positron emission tomography: role in a multimodality approach. *J Comput Assist Tomogr* **2003**, *27*, 70-77, doi: 10.1097/00004728-200301000-00013.
6. Sato, N.; Inoue, T.; Tomiyoshi, K.; Aoki, J.; Oriuchi, N.; Takahashi, A.; Otani, T.; Kurihara, H.; Sasaki, T.; Endo, K. Gliomatosis cerebri evaluated by ^{18}F-α-methyl tyrosine positron-emission tomography. *Neuroradiology* **2003**, *45*, 700-707, doi: 10.1007/s00234-003-1057-2.
7. Suzuki, H.; Watanabe, H.; Shinozaki, T.; Yanagawa, T.; Suzuki, R.; Takagishi, K. Positron emission tomography imaging of musculoskeletal tumors in the shoulder girdle. *J Shoulder Elbow Surg* **2004**, *13*, 635-647, doi: 10.1016/S1058274604001314.
8. Suzuki, R.; Watanabe, H.; Yanagawa, T.; Sato, J.; Shinozaki, T.; Suzuki, H.; Endo, K.; Takagishi, K. PET evaluation of fatty tumors in the extremity: Possibility of using the standardized uptake value (SUV) to differentiate benign tumors from liposarcoma. *Ann Nucl Med* **2005**, *19*, 661-670, doi: 10.1007/BF02985114.
9. Higuchi, T.; Oriuchi, N.; Miyakubo, M.; Ishikita, T.; Otake, H.; Koyama, K.; Endo, K. Preliminary evaluation of Fluorine-18 alpha-methyl tyrosine PET in the differential diagnosis of pancreatic tumor; comparative study with FDG-PET. In Proceedings of Annual Congress of the EANM 2006, Athens; p. S257.
10. Kaira, K.; Oriuchi, N.; Otani, Y.; Yanagitani, N.; Sunaga, N.; Hisada, T.; Ishizuka, T.; Endo, K.; Mori, M. Diagnostic usefulness of fluorine-18-alpha-methyltyrosine positron emission tomography in combination with ^{18}F-fluorodeoxyglucose in sarcoidosis patients. *Chest* **2007**, *131*, 1019-1027, doi: 10.1378/chest.06-2160.
11. Kaira, K.; Oriuchi, N.; Otani, Y.; Shimizu, K.; Tanaka, S.; Imai, H.; Yanagitani, N.; Sunaga, N.; Hisada, T.; Ishizuka, T., et al. Fluorine-18-alpha-methyltyrosine positron emission tomography for diagnosis and staging of lung cancer: a clinicopathologic study. *Clin Cancer Res* **2007**, *13*, 6369-6378, doi: 10.1158/1078-0432.CCR-07-1294.
12. Miyakubo, M.; Oriuchi, N.; Tsushima, Y.; Higuchi, T.; Koyama, K.; Arai, K.; Paudyal, B.; Iida, Y.; Hanaoka, H.; Ishikita, T., et al. Diagnosis of maxillofacial tumor with L-3-[^{18}F]-fluoro-alpha-methyltyrosine (FMT) PET: a comparative study with FDG-PET. *Ann Nucl Med* **2007**, *21*, 129-135, doi: doi:10.1007/BF03033991.
13. Kaira, K.; Oriuchi, N.; Shimizu, K.; Ishikita, T.; Higuchi, T.; Imai, H.; Yanagitani, N.; Sunaga, N.; Hisada, T.; Ishizuka, T., et al. Evaluation of thoracic tumors with ^{18}F-FMT and ^{18}F-FDG PET-CT: a clinicopathological study. *Int J Cancer* **2009**, *124*, 1152-1160, doi: 10.1002/ijc.24034.
14. Kaira, K.; Oriuchi, N.; Shimizu, K.; Ishikita, T.; Higuchi, T.; Imai, H.; Yanagitani, N.; Sunaga, N.; Hisada, T.; Ishizuka, T., et al. Correlation of angiogenesis with ^{18}F-FMT and ^{18}F-FDG uptake in non-small cell lung cancer. *Cancer Sci* **2009**, *100*, 753-758, doi: 10.1111/j.1349-7006.2008.01077.x.
15. Kaira, K.; Oriuchi, N.; Shimizu, K.; Tominaga, H.; Yanagitani, N.; Sunaga, N.; Ishizuka, T.; Kanai, Y.; Mori, M.; Endo, K. ^{18}F-FMT Uptake Seen Within Primary Cancer on PET Helps Predict Outcome of Non-Small Cell Lung Cancer. *J Nucl Med* **2009**, *50*, 1770-1776, doi: 10.2967/jnumed.109.066837.
16. Nakano, S.; Oriuchi, N.; Higuchi, T.; Arisaka, Y.; Endo, K. Initial evaluation of [F-18] alpha-methyl tyrosine PET for differential diagnosis of pancreatic lesions. In Proceedings of SNM 2009 Annual Meeting, Toronto, Canada, June 16 2009; 50, *2(Supp.)*, p. 1753. Online abstract: http://jnm.snmjournals.org/content/50/supplement_2/1753.abstract
17. Kaira, K.; Oriuchi, N.; Yanagitani, N.; Sunaga, N.; Ishizuka, T.; Mori, M.; Endo, K. Assessment of therapy response in lung cancer with ^{18}F-α-methyl tyrosine PET. *AJR Am J Roentgenol* **2010**, *195*, 1204-1211, doi: 10.2214/AJR.09.4167.
18. Kaira, K.; Oriuchi, N.; Shimizu, K.; Imai, H.; Tominaga, H.; Yanagitani, N.; Sunaga, N.; Hisada, T.; Ishizuka, T.; Kanai, Y., et al. Comparison of L-type amino acid transporter 1 expression and L-[3-^{18}F]-α-methyl tyrosine uptake in outcome of non-small cell lung cancer. *Nucl Med Biol* **2010**, *37*, 911-916, doi: 10.1016/j.nucmedbio.2010.06.004.
19. Miyashita, G.; Higuchi, T.; Oriuchi, N.; Arisaka, Y.; Hanaoka, H.; Tominaga, H.; Morita, S.; Miyakubo, M.; Ishikita, T.; Nakasone, Y., et al. ^{18}F-FAMT uptake correlates with tumor proliferative activity in oral squamous cell carcinoma: comparative study with ^{18}F-FDG PET and immunohistochemistry. *Ann Nucl Med* **2010**, *24*, 579-584, doi: 10.1007/s12149-010-0398-2.
20. Oriuchi, N.; Kaira, K.; Shimizu, K.; Tominaga, H.; Hanaoka, H.; Arisaka, Y.; Higuchi, T.; Naoe, T.; Endo, K. Clinical significance of ^{18}F-alpha-methyl tyrosine PET as a potential surrogate marker of LAT1 expression for predicting prognosis of non-small cell lung cancer. *J Clin Oncol, 2010 ASCO Annual Meeting Abstracts* **2010**, *28*, 10559.
21. Sohda, M.; Kato, H.; Suzuki, S.; Tanaka, N.; Sano, A.; Sakai, M.; Inose, T.; Nakajima, M.; Miyazaki, T.; Fukuchi, M., et al. ^{18}F-FAMT-PET is useful for the diagnosis of lymph node metastasis in operable esophageal squamous cell carcinoma. *Ann Surg Oncol* **2010**, *17*, 3181-3186, doi: 10.1245/s10434-010-1177-y.
22. Tian, M.; Zhang, H.; Endo, K. Comparison of cell proliferation, protein, and glucose metabolism in musculoskeletal tumors in a PET study. *J Biomed Biotechnol* **2011**, *2011*, 807929, doi: 10.1155/2011/807929.

Supplementary Table S2. LAT1-specific PET radiopharmaceuticals in clinical studies (in comparison with [^{18}F]FDG)

23. Isoda, A.; Higuchi, T.; Nakano, S.; Arisaka, Y.; Kaira, K.; Kamio, T.; Mawatari, M.; Matsumoto, M.; Sawamura, M.; Tsushima, Y. ^{18}F-FAMT in patients with multiple myeloma: Clinical utility compared to ^{18}F-FDG. *Ann Nucl Med* **2012**, *26*, 811-816, doi: 10.1007/s12149-012-0645-9.
24. Morita, M.; Higuchi, T.; Achmad, A.; Tokue, A.; Arisaka, Y.; Tsushima, Y. Complementary roles of tumour specific PET tracer ^{18}F-FAMT to ^{18}F-FDG PET/CT for the assessment of bone metastasis. *Eur J Nucl Med Mol Imaging* **2013**, *40*, 1672-1681, doi: 10.1007/s00259-013-2487-7.
25. Kim, M.; Higuchi, T.; Arisaka, Y.; Achmad, A.; Tokue, A.; Tominaga, H.; Miyashita, G.; Miyazaki, H.; Negishi, A.; Yokoo, S., et al. Clinical significance of ^{18}F-α-methyl tyrosine PET/CT for the detection of bone marrow invasion in patients with oral squamous cell carcinoma: comparison with ^{18}F-FDG PET/CT and MRI. *Ann Nucl Med* **2013**, *27*, 423-430, doi: 10.1007/s12149-013-0701-0.
26. Nobusawa, A.; Kim, M.; Kaira, K.; Miyashita, G.; Negishi, A.; Oriuchi, N.; Higuchi, T.; Tsushima, Y.; Kanai, Y.; Yokoo, S., et al. Diagnostic usefulness of ^{18}F-FAMT PET and L-type amino acid transporter 1 (LAT1) expression in oral squamous cell carcinoma. *Eur J Nucl Med Mol Imaging* **2013**, *40*, 1692-1700, doi: 10.1007/s00259-013-2477-9.
27. Sohda, M.; Miyazaki, T.; Honjyo, H.; Hara, K.; Ozawa, D.; Suzuki, S.; Tanaka, N.; Sano, A.; Sakai, M.; Yokobori, T., et al. L-[3-^{18}F]-α-methyltyrosine uptake by lymph node metastasis is a predictor of complete response to CRT in esophageal cancer. *Anticancer Res* **2014**, *34*, 7473-7477.
28. Sohda, M.; Honjyo, H.; Hara, K.; Ozawa, D.; Suzuki, S.; Tanaka, N.; Sano, A.; Sakai, M.; Yokobori, T.; Inose, T., et al. L-[3-^{18}F]-α-Methyltyrosine Accumulation as a Definitive Chemoradiotherapy Response Predictor in Patients with Esophageal Cancer. *Anticancer Res* **2014**, *34*, 909-913, doi: 34/2/909.
29. Sohda, M.; Sakai, M.; Honjyo, H.; Hara, K.; Ozawa, D.; Suzuki, S.; Tanaka, N.; Yokobori, T.; Miyazaki, T.; Fukuchi, M., et al. Use of pre-treatment ^{18}F-FAMT PET to predict patient survival in squamous cell carcinoma of the esophagus treated by curative surgery. *Anticancer Res* **2014**, *34*, 3623-3628.
30. Suzuki, S.; Kaira, K.; Ohshima, Y.; Ishioka, N.S.; Sohda, M.; Yokobori, T.; Miyazaki, T.; Oriuchi, N.; Tominaga, H.; Kanai, Y., et al. Biological significance of fluorine-18-α-methyltyrosine (FAMT) uptake on PET in patients with oesophageal cancer. *Br J Cancer* **2014**, *110*, 1985-1991, doi: 10.1038/bjc.2014.142.
31. Kim, M.; Achmad, A.; Higuchi, T.; Arisaka, Y.; Yokoo, H.; Yokoo, S.; Tsushima, Y. Effects of Intratumoral Inflammatory Process on ^{18}F-FDG Uptake: Pathologic and Comparative Study with ^{18}F-Fluoro-α-Methyltyrosine PET/CT in Oral Squamous Cell Carcinoma. *J Nucl Med* **2015**, *56*, 16-21, doi: 10.2967/jnumed.114.144014.
32. Kodaira, S.; Nakajima, T.; Arisaka, Y.; Tokue, A.; Higuchi, T.; Tsushima, Y. Advantages of L-3-[^{18}F] fluoro-alpha-methyl tyrosine over 2-[^{18}F]-fluoro-2-deoxyglucose in detecting liver metastasis during positron emission tomography scan. *SpringerPlus* **2016**, *5*, 618, doi: 10.1186/s40064-016-2212-7.
33. Kaira, K.; Higuchi, T.; Sunaga, N.; Arisaka, Y.; Hisada, T.; Tominaga, H.; Oriuchi, N.; Asao, T.; Tsushima, Y.; Yamada, M. Usefulness of ^{18}F-α-Methyltyrosine PET for Therapeutic Monitoring of Patients with Advanced Lung Cancer. *Anticancer Research* **2016**, *36*, 6481-6490, doi: 10.21873/anticanres.11247.
34. Horiguchi, K.; Tosaka, M.; Higuchi, T.; Arisaka, Y.; Sugawara, K.; Hirato, J.; Yokoo, H.; Tsushima, Y.; Yoshimoto, Y. Clinical value of fluorine-18 α-methyltyrosine PET in patients with gliomas: comparison with fluorine-18 fluorodeoxyglucose PET. *EJNMMI research* **2017**, *7*, 1-10, doi: 10.1186/s13550-017-0298-8.
35. Honjo, H.; Toh, Y.; Sohda, M.; Suzuki, S.; Kaira, K.; Kanai, Y.; Nagamori, S.; Oyama, T.; Yokobori, T.; Miyazaki, T., et al. Clinical Significance and Phenotype of MTA1 Expression in Esophageal Squamous Cell Carcinoma. *Anticancer Research* **2017**, *37*, 4147-4155, doi: 10.21873/anticanres.11802.
36. Sohda, M.; Miyazaki, T.; Honjyo, H.; Hara, K.; Ozawa, D.; Sakai, M.; Yokobori, T.; Higuchi, T.; Tsushima, Y.; Kuwano, H. ^{18}F-FAMT PET Is Useful to Distinguish between Specific Uptake and Nonspecific Uptake Compared to ^{18}F-Flourodeoxyglucose Position Emission Tomography in Esophageal Cancer Patients. *Digestive Surgery* **2017**, *35*, 383-388, doi: 10.1159/000480122.
37. Sohda, M.; Honjyo, H.; Hara, K.; Ozawa, D.; Suzuki, S.; Tanaka, N.; Sano, A.; Sakai, M.; Inose, T.; Miyazaki, T., et al. ^{18}F-FAMT-PET Is Useful for Judging Clinical Complete Response in Advanced Esophageal Cancer Patients Who Have Received Definitive Chemoradiotherapy. *International Surgery* **2018**, *103*, 561-566, doi: 10.9738/intsurg-d-16-00163.1.
38. Kumasaka, S.; Nakajima, T.; Arisaka, Y.; Tokue, A.; Achmad, A.; Fukushima, Y.; Shimizu, K.; Kaira, K.; Higuchi, T.; Tsushima, Y. Prognostic value of metabolic tumor volume of pretreatment ^{18}F-FAMT PET/CT in non-small cell lung Cancer. *BMC Medical Imaging* **2018**, *18*, 46, doi: 10.1186/s12880-018-0292-2.
39. Kim, M.; Higuchi, T.; Nakajima, T.; Andriana, P.; Hirasawa, H.; Tokue, A.; Kurihara, J.; Yokoo, S.; Tsushima, Y. ^{18}F-FDG and ^{18}F-FAMT PET-derived metabolic parameters predict outcome of oral squamous cell carcinoma. *Oral Radiol* **2019**, *35*, 308-314, doi: 10.1007/s11282-019-00377-2.
40. Kuriyama, K.; Higuchi, T.; Yokobori, T.; Saito, H.; Yoshida, T.; Hara, K.; Suzuki, S.; Sakai, M.; Sohda, M.; Higuchi, T., et al. Uptake of positron emission tomography tracers reflects the tumor immune status in esophageal squamous cell carcinoma. *Cancer Science* **2020**, *111*, 1969-1978, doi: 10.1111/cas.14421.
41. Shimizu, K.; Kaira, K.; Higuchi, T.; Hisada, T.; Yokobori, T.; Oyama, T.; Asao, T.; Tsushima, Y.; Shirabe, K. Relationship Between Tumor Immune Markers and Fluorine-18-α-Methyltyrosine ([^{18}F]FAMT) Uptake in Patients with Lung Cancer. *Molecular Imaging and Biology* **2020**, *22*, 1078-1086, doi: 10.1007/s11307-019-01456-w.
42. Suzuki, K.; Kurihara, J.; Kim, M.; Yanagisawa, S.; Ogawa, M.; Makiguchi, T.; Yokoo, S. Image screening for maxillo-mandibular actinomycosis with CT, ^{18}F-FDG-PET/CT, and ^{18}F-α-methyl tyrosine PET/CT. *Oral Radiology* **2021**, *37*, 46-54, doi: 10.1007/s11282-020-00421-6.
43. Yoshino, E.; Ohmori, Y.; Imahori, Y.; Higuchi, T.; Furuya, S.; Naruse, S.; Mori, T.; Suzuki, K.; Yamaki, T.; Ueda, S., et al. Irradiation Effects on the Metabolism of Metastatic Brain Tumors: Analysis by Positron Emission Tomography and ^{1}H-Magnetic Resonance Spectroscopy. *Stereotactic and Functional Neurosurgery* **1997**, *66*, 240-259, doi: 10.1159/000099816.

Supplementary Table S2. LAT1-specific PET radiopharmaceuticals in clinical studies (in comparison with [^{18}F]FDG)

44. Mishima, Y.; Imahori, Y.; Honda, C.; Hiratsuka, J.; Ueda, S.; Ido, T. In vivo diagnosis of human malignant melanoma with positron emission tomography using specific melanoma-seeking ^{18}F-DOPA analogue. *Journal of Neuro-Oncology* **1997**, *33*, 163-169, doi: 10.1023/A:1005746020350.
45. Kabalka, G.W.; Smith, G.T.; Dyke, J.P.; Reid, W.S.; Longford, C.P.D.; Roberts, T.G.; Reddy, N.K.; Buonocore, E.; Hübner, K.F. Evaluation of Fluorine-18-BPA-Fructose for Boron Neutron Capture Treatment Planning. *Journal of Nuclear Medicine* **1997**, *38*, 1762.
46. Imahori, Y.; Ueda, S.; Ohmori, Y.; Kusuki, T.; Ono, K.; Fujii, R.; Ido, T. Fluorine-18-labeled fluoroboronophenylalanine PET in patients with glioma. *J Nucl Med* **1998**, *39*, 325-333.
47. Takahashi, Y.; Imahori, Y.; Mineura, K. Prognostic and therapeutic indicator of fluoroboronophenylalanine positron emission tomography in patients with gliomas. *Clin Cancer Res* **2003**, *9*, 5888-5895.
48. Havu-Aurén, K.; Kiiski, J.; Lehtiö, K.; Eskola, O.; Kulvik, M.; Vuorinen, V.; Oikonen, V.; Vähätalo, J.; Jääskeläinen, J.; Minn, H. Uptake of 4-borono-2-[^{18}F]fluoro-L-phenylalanine in sporadic and neurofibromatosis 2-related schwannoma and meningioma studied with PET. *Eur J Nucl Med Mol Imaging* **2007**, *34*, 87-94, doi: 10.1007/s00259-006-0154-y.
49. Miyatake, S.-I.; Tamura, Y.; Kawabata, S.; Iida, K.; Kuroiwa, T.; Ono, K. Boron neutron capture therapy for malignant tumors related to meningiomas. *Neurosurgery* **2007**, *61*, 82-91, doi: 10.1227/01.neu.0000279727.90650.24.
50. Ariyoshi, Y.; Miyatake, S.-I.; Kimura, Y.; Shimahara, T.; Kawabata, S.; Nagata, K.; Suzuki, M.; Maruhashi, A.; Ono, K.; Shimahara, M. Boron neuron capture therapy using epithermal neutrons for recurrent cancer in the oral cavity and cervical lymph node metastasis. *Oncol Rep* **2007**, *18*, 861-866, doi: 10.3892/or.18.4.861.
51. Kankaanranta, L.; Seppälä, T.; Koivunoro, H.; Saarilahti, K.; Atula, T.; Collan, J.; Salli, E.; Kortesniemi, M.; Uusi-Simola, J.; Mäkitie, A. Boron neutron capture therapy in the treatment of locally recurred head and neck cancer. *International Journal of Radiation Oncology* Biology* Physics* **2007**, *69*, 475-482.
52. Fuwa, N.; Suzuki, M.; Sakurai, Y.; Nagata, K.; Kinashi, Y.; Masunaga, S.; Maruhashi, A.; Imahori, Y.; Kodaira, T.; Tachibana, H., et al. Treatment results of boron neutron capture therapy using intra-arterial administration of boron compounds for recurrent head and neck cancer. *British Journal of Radiology* **2014**, *81*, 749-752, doi: 10.1259/bjr/65306248.
53. Nariai, T.; Ishiwata, K.; Kimura, Y.; Inaji, M.; Momose, T.; Yamamoto, T.; Matsumura, A.; Ishii, K.; Ohno, K. PET pharmacokinetic analysis to estimate boron concentration in tumor and brain as a guide to plan BNCT for malignant cerebral glioma. *Applied Radiation and Isotopes* **2009**, *67*, S348-S350, doi: https://doi.org/10.1016/j.apradiso.2009.03.061.
54. Kimura, Y.; Ariyoshi, Y.; Shimahara, M.; Miyatake, S.; Kawabata, S.; Ono, K.; Suzuki, M.; Maruhashi, A. Boron neutron capture therapy for recurrent oral cancer and metastasis of cervical lymph node. *Applied Radiation and Isotopes* **2009**, *67*, S47-S49, doi: https://doi.org/10.1016/j.apradiso.2009.03.019.
55. Kankaanranta, L.; Seppälä, T.; Koivunoro, H.; Saarilahti, K.; Atula, T.; Collan, J.; Salli, E.; Kortesniemi, M.; Uusi-Simola, J.; Välimäki, P., et al. Boron Neutron Capture Therapy in the Treatment of Locally Recurred Head-and-Neck Cancer: Final Analysis of a Phase I/II Trial. *International Journal of Radiation Oncology, Biology, Physics* **2012**, *82*, e67-e75, doi: 10.1016/j.ijrobp.2010.09.057.
56. Kawabata, S.; Hiramatsu, R.; Kuroiwa, T.; Ono, K.; Miyatake, S. Boron neutron capture therapy for recurrent high-grade meningiomas. *J Neurosurg* **2013**, *119*, 837-844, doi: 10.3171/2013.5.Jns122204.
57. Tani, H.; Kurihara, H.; Hiroi, K.; Honda, N.; Yoshimoto, M.; Kono, Y.; Murakami, R.; Kumita, S.; Arai, Y.; Itami, J. Correlation of ^{18}F-BPA and ^{18}F-FDG uptake in head and neck cancers. *Radiother Oncol* **2014**, *113*, 193-197, doi: 10.1016/j.radonc.2014.11.001.
58. Kobayashi, K.; Kurihara, H.; Watanabe, Y.; Murakami, N.; Inaba, K.; Nakamura, S.; Wakita, A.; Okamoto, H.; Umezawa, R.; Takahashi, K., et al. In vivo spatial correlation between ^{18}F-BPA and ^{18}F-FDG uptakes in head and neck cancer. *Applied Radiation and Isotopes* **2016**, *115*, 138-146, doi: https://doi.org/10.1016/j.apradiso.2016.05.026.
59. Watabe, T.; Ikeda, H.; Nagamori, S.; Wiriyasermkul, P.; Tanaka, Y.; Naka, S.; Kanai, Y.; Hagiwara, K.; Aoki, M.; Shimosegawa, E. ^{18}F-FBPA as a tumor-specific probe of L-type amino acid transporter 1 (LAT1): a comparison study with ^{18}F-FDG and ^{11}C-Methionine PET. *Eur J Nucl Med Mol Imaging* **2017**, *44*, 321-331, doi: 10.1007/s00259-016-3487-1.
60. Morita, T.; Kurihara, H.; Hiroi, K.; Honda, N.; Igaki, H.; Hatazawa, J.; Arai, Y.; Itami, J. Dynamic changes in ^{18}F-borono-L-phenylalanine uptake in unresectable, advanced, or recurrent squamous cell carcinoma of the head and neck and malignant melanoma during boron neutron capture therapy patient selection. *Radiation Oncology* **2018**, *13*, 4, doi: 10.1186/s13014-017-0949-y.
61. Beshr, R.; Isohashi, K.; Watabe, T.; Naka, S.; Horitsugi, G.; Romanov, V.; Kato, H.; Miyatake, S.-I.; Shimosegawa, E.; Hatazawa, J. Preliminary feasibility study on differential diagnosis between radiation-induced cerebral necrosis and recurrent brain tumor by means of [^{18}F]fluoro-borono-phenylalanine PET/CT. *Annals of Nuclear Medicine* **2018**, *32*, 702-708, doi: 10.1007/s12149-018-1296-2.
62. Igaki, H.; Nakamura, S.; Kurihara, H.; Abe, Y.; Nishioka, S.; Fujii, R.; Nakamura, M.; Nakayama, Y.; Morita, T.; Okamoto, H., et al. Comparison of ^{18}FBPA uptake with ^{18}FDG uptake in cancer patients. *Appl Radiat Isot* **2020**, *157*, 109019, doi: 10.1016/j.apradiso.2019.109019.
63. Lo, Y.-W.; Lee, J.-C.; Hu, Y.-S.; Li, C.-Y.; Chen, Y.-L.; Lin, C.-S.; Huang, W.-S.; Lin, K.-H.; Chen, Y.-W. The importance of optimal ROIs delineation for FBPA-PET before BNCT. *Applied Radiation and Isotopes* **2020**, *163*, 109219, doi: https://doi.org/10.1016/j.apradiso.2020.109219.
64. Isohashi, K.; Kanai, Y.; Aihara, T.; Hu, N.; Fukushima, K.; Baba, I.; Hirokawa, F.; Kakino, R.; Komori, T.; Nihei, K., et al. Exploration of the threshold SUV for diagnosis of malignancy using ^{18}F-FBPA PET/CT. *Eur J Hybrid Imaging* **2022**, *6*, 35, doi: 10.1186/s41824-022-00156-z.

Supplementary Table S2. LAT1-specific PET radiopharmaceuticals in clinical studies (in comparison with [^{18}F]FDG)

65. Minoji, T.; Takahashi, H.; Watabe, T.; Ishikawa, S.; Kato, H.; Ohgaki, R.; Kanai, Y.; Sekido, Y.; Hata, T.; Hamabe, A. The Clinical Potential of FBPA-PET/CT Imaging for Colorectal Cancer and its Liver Metastases Expressing LAT1. *Anticancer Research* **2023**, *43*, 3913-3921.
66. Watabe, T.; Ose, N.; Naka, S.; Fukui, E.; Kimura, T.; Kanou, T.; Funaki, S.; Sasaki, H.; Kamiya, T.; Kurimoto, K., et al. Evaluation of LAT1 Expression in Patients with Lung Cancer and Mediastinal Tumors: ^{18}F-FBPA PET Study with Immunohistological Comparison. *Clinical Nuclear Medicine* **2023**, *48*, 853-860, doi: 10.1097/rlu.0000000000004816.
67. Li, Z.; Kong, Z.; Chen, J.; Li, J.; Li, N.; Yang, Z.; Wang, Y.; Liu, Z. ^{18}F-Boramino acid PET/CT in healthy volunteers and glioma patients. *Eur J Nucl Med Mol Imaging* **2021**, *48*, 3113-3121, doi: 10.1007/s00259-021-05212-7.
68. Kong, Z.; Li, Z.; Chen, J.; Liu, S.; Liu, D.; Li, J.; Li, N.; Ma, W.; Feng, F.; Wang, Y. Metabolic characteristics of [^{18}F] fluoroboronotyrosine (FBY) PET in malignant brain tumors. *Nuclear Medicine and Biology* **2022**, *106*, 80-87.
69. Kong, Z.; Li, Z.; Chen, J.; Ma, W.; Wang, Y.; Yang, Z.; Liu, Z. Larger ^{18}F-fluoroboronotyrosine (FBY) active volume beyond MRI contrast enhancement in diffuse gliomas than in circumscribed brain tumors. *EJNMMI Res* **2022**, *12*, 22, doi: 10.1186/s13550-022-00896-w.
